# Assessing changing baleen whale distributions and reported incidents relative to vessel activity in the Northwest Atlantic

**DOI:** 10.1371/journal.pone.0315909

**Published:** 2025-01-15

**Authors:** Hannah Solway, Boris Worm, Tonya Wimmer, Derek P. Tittensor

**Affiliations:** 1 Department of Biology, Dalhousie University, Halifax, NS, Canada; 2 Marine Animal Response Society, Halifax, NS, Canada; Swedish University of Agricultural Sciences and Swedish Institute for the Marine Environment, University of Gothenburg, SWEDEN

## Abstract

Baleen whales are among the largest marine megafauna, and while mostly well-protected from direct exploitation, they are increasingly affected by vessel traffic, interactions with fisheries, and climate change. Adverse interactions, notably vessel strikes and fishing gear entanglement, often result in distress, injury, or death for these animals. In Atlantic Canadian waters, such negative interactions or ‘incidents’ are consistently reported to marine animal response organizations but have not yet been analyzed relative to the spatial distribution of whales and vessels. Using a database of 483,003 whale sightings, 1,110 incident reports, and 82 million hours of maritime vessel activity, we conducted a spatiotemporal vulnerability analysis for all six baleen whale species occurring in the Northwest Atlantic Ocean by developing an ensemble of habitat-suitability models. The relative spatial risk of vessel-induced incidents was assessed for present (1985–2015) and projected near-future (2035–2055) distributions of baleen whales. Areas of high habitat suitability for multiple baleen whale species were intrinsically linked to sea surface temperature and salinity, with multispecies hotspots identified in the Bay of Fundy, Scotian Shelf, Laurentian Channel, Flemish Cap, and Gulf of St. Lawrence. Present-day model projections were independently evaluated using a separate database of acoustic detections and found to align well. Regions of high relative incident risk were projected close to densely inhabited regions, principal maritime routes, and major fishing grounds, in general coinciding with reported incident hotspots. While some high-risk regions already benefit from mitigation strategies aimed at protecting North Atlantic Right Whales, our analysis highlights the importance of considering risks to multiple species, both in the present day and under continued environmental change.

## Introduction

The Northwest Atlantic Ocean (NWA) is home to six baleen whale species: blue (*Balaenoptera musculus*), fin (*Balaenoptera physalus*), humpback (*Megaptera novaeangliae*), minke (*Balaenoptera acutorostrata*), North Atlantic right (*Eubalena glacialis*; herein referred to as NA right), and sei whales (*Balaenoptera borealis*). These species all have previously declined in abundance due to commercial whaling [1,2]. Fortunately, conservation efforts over recent decades, including the 1986 International Whaling Commission moratorium and protective policies throughout the US and Canada, have enabled recovery in some species, most notably humpbacks [2]. Other species, such as blue, fin and NA right whales are still threatened [2–5], in stark contrast to some resurgent southern Atlantic populations [6–9].

**Fig 1:**
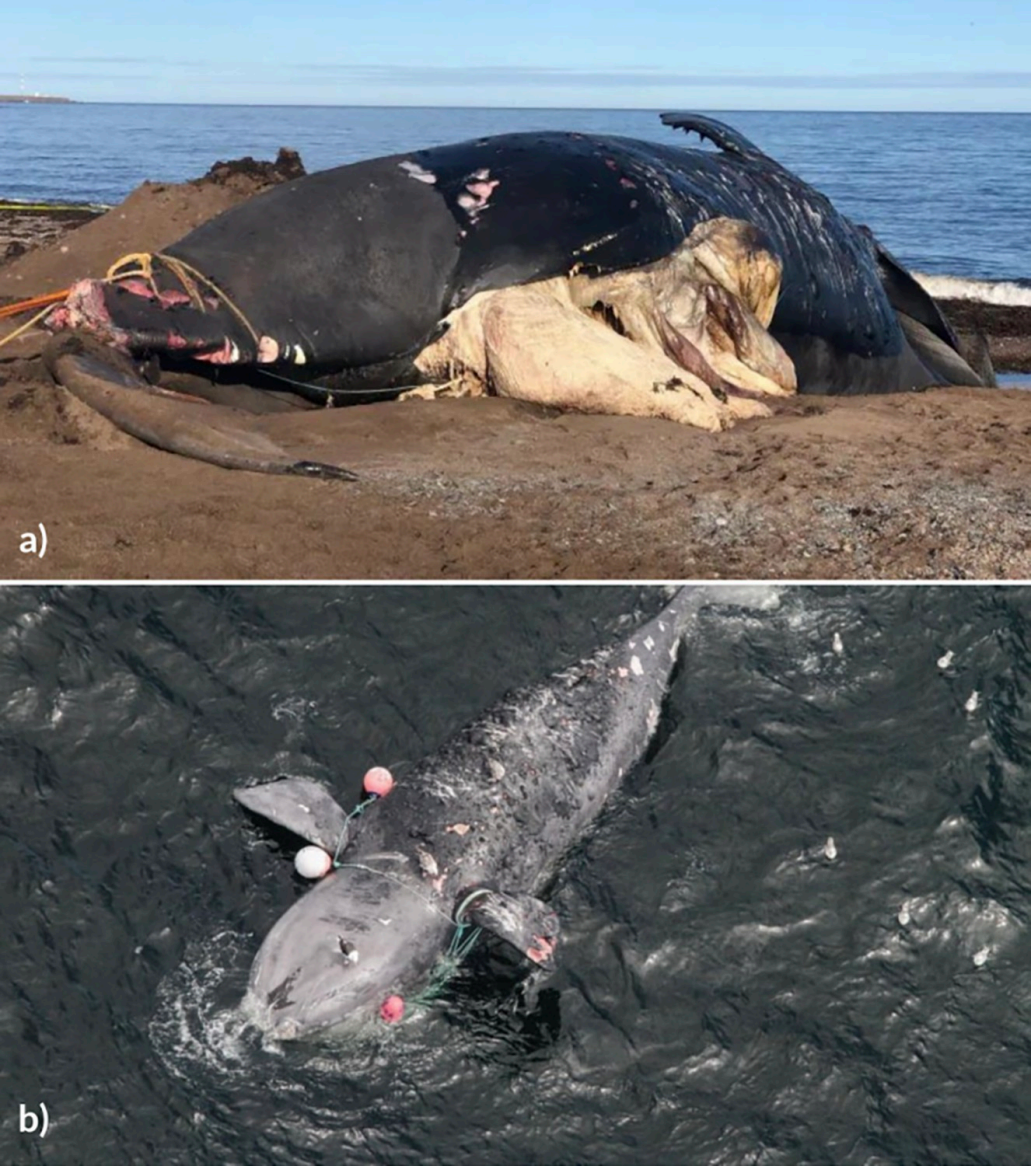
Major risks to baleen whales. Examples of NA right whales killed by (a) ship strikes (b) Entanglement in fishing gear. Image credits: (a) Marine Animal Response Society, collected under federal SARA permit issued to MARS. (b) NEFSC taken under SARA Permit DFO-MAR-2016-02 (Amendment 1) and NMFS Permit 17355.

Increasing threats from motorized vessel activity, fishing gear entanglements, and climate change are thought to jeopardize baleen whale recovery in the NWA region (Fig 1) [10]. The NA right whale’s population is particularly depleted, with fewer than 75 breeding females remaining globally [11–13]. Between 1970 and 2006, 53% of studied NA right whales in the NWA fell victim to vessel strikes [12,14], along with 30% of humpbacks [15]. Moreover, 83% of NA right whales show scars from entanglements [16]. Furthermore, there is potential underreporting in such statistics, particularly for individuals that sink upon death [17].

When baleen whale incidents such as entanglements or vessel strikes in the NWA are observed (Fig 1), dedicated marine animal response organizations are typically notified [18]. Data from 2004 to 2019 suggest that, of those animals where the cause of death was determined, entanglements caused 46% of documented fatalities, with minke whales most commonly reported [18,19]. Vessel strikes represented 15% of determined causes of death, with NA right whales disproportionately affected, especially during unprecedented mass mortality events in 2017 and 2019 [18,19].

Common traits among baleen whales such as delayed sexual maturity, lengthy gestation periods, and extensive calving intervals account for their slow recovery from present or past adversities [2,20]. While individual species exhibit a range of sizes and physiological characteristics, they generally share similar diets–large zooplankton and in some cases schooling forage fish—and comparable migratory patterns [21–27].

More recently, climate change is reshaping baleen whale distributions, predominantly through alterations in prey availability tied to changing ocean temperatures [11,28]. Climate-induced changes in food sources have driven NA right whales further north to the Gulf of St. Lawrence, making these species vulnerable to vessel strikes and entanglements in this region [10,11,29–32]. Concurrently, other baleen whale species are likely facing analogous challenges, stressing the need for updated distribution information and an adaptive approach to their spatial protection [33].

Four of six NWA baleen whale species are currently listed or under consideration for listing as endangered (blue, NA right, and sei whales) or of special concern (the fin whale) under the Species at Risk Act (SARA), Canada’s official framework for safeguarding threatened wildlife [34]. In response to the 2017 and 2019 NA right whale mass mortality events, Fisheries and Oceans Canada (DFO) and Transport Canada (TC) have implemented both voluntary and mandatory protective regulations to minimize vessel strikes and entanglements for right whales, such as distance-keeping and slow-down measures. Here, NA right whale sightings and acoustic detections have informed the placement of spatio-temporal fishing closures and vessel slowdowns to protect this species where it is currently observed [35,36].

While there has been a reduction in reported mortalities of NA right whales in Canada since the instatement of slowdown measures and fishery closures [37], their efficacy for other whale species is unknown [38]. Furthermore, an understanding of how such measures might need to change as baleen whale distributions shift over the coming century remains elusive [39,40].

While there have been regional efforts to reduce incidents and promote population recovery, there remains a critical need for better information to inform management decisions. This includes understanding where incidents are likely to occur, and the relationship between incident risk, vessel traffic, and whale distributions. Knowledge of vessel traffic patterns, at least for larger vessels equipped with Automatic Identification System (AIS) transponders, is reasonably well-established [10,41]. However, our understanding of whale distributions in the region remains limited due to incomplete observer coverage or ineffective methods to detect whales. To address this knowledge gap and feed into a more comprehensive assessment of risk, species distribution models (SDMs) have emerged as valuable tools to project baleen whale habitat use by examining their relationship with leading environmental variables such as temperature, productivity and habitat, among others [26,29,39,40,42,43]. When coupled with projections of future climate conditions, SDMs can help project how baleen whale habitat suitability will change over time [39], providing important context for long-term recovery plans [44].

The core objectives of this study are to (1) deepen our understanding of the potential for interactions between vessels and baleen whales in the NWA, (2) to project areas of high risk for whale incidents, and (3) to evaluate how climate change may affect the distribution of baleen whales and incident risk over time. We do this by integrating whale sighting and vessel databases to conduct a spatiotemporal risk analysis for all six baleen whale species, with species distribution models (SDMs) as our primary tool. By doing so, we aim to generate insights that can help to strengthen existing management strategies across different baleen whale species in the Northwest Atlantic.

## Materials and methods

### Study area

The study region encompasses Canada’s east coast in the Northwest Atlantic, with a specific focus on five distinct areas: the Laurentian Channel, Bay of Fundy, Scotian Shelf, Gulf of St Lawrence, and coastal, shelf, and offshore Newfoundland and southern Labrador.

### Incident data

The Marine Animal Response Society (MARS) compiled data regarding baleen whale incidents reported to emergency hotlines on the east coast of Canada between 2004 and 2019 [18,19]. In total, data on 1,110 baleen whale incidents were provided for this study by MARS as compiled from the Maritime Provinces (MARS), Newfoundland and Labrador (Whale Release and Strandings) and Quebec (Quebec Marine Mammal Emergency Network) [19]. An "incident" involving baleen whales in the NWA is defined as any reported animal that is found in distress, injured, or dead. At a minimum, each incident report contained the species (where known), the location the animal was observed, as well as the date and details on the animal’s behaviour and condition (e.g., entangled, injured, or deceased). In most cases, the cause of the incident was not discernible, including whether it was caused by an anthropogenic source, such as entanglement, vessel strike, or ingestion of marine debris. Incidents were reported to hotlines from a variety of sources, including coastal observations and from aerial- and ship-based platforms. Species identification, where possible, was conducted by trained experts [19]. No sampling effort or incident absence data were available for this database.

Incidents were primarily reported in the spring and summer, with fewer reports from the fall and winter months. Humpback and minke whale incidents were most frequently reported, while fin, NA right, blue, and sei whales had fewer incidents [18,19]. Most of the reports described deceased whales, while a small number did not specify the whale’s condition. The incidents were also classified into several categories, with entanglements, beached carcasses, and floating carcasses being the most common [18,19].

We note that most incidents are likely to be observed with some time lag and may only be detected once the whales travel or drift near to shore where they are more commonly observed [18,19]. As such, the date and location when an animal was reported may not reflect precisely when or where the incident actually occurred. Given these uncertainties, point incident data were aggregated over a larger 1° x 1° grid spanning the NWA using QGIS vector geometry methods [45]. The number of incident reports per 1° grid cell across years was then calculated for individual species and across all baleen whales combined. All subsequent vessel activity and opportunistic sightings data processing and averaging were carried out in QGIS using the same methods.

### Vessel data

Vessel activity data from 2017–2021 were sourced from Global Fishing Watch (GFW) via data generated by Automatic Identification System (AIS) transponders aboard vessels [41]. While this coverage is substantial (82,141,732 hours of vessel observations in the region of interest over the study period), it is limited to vessels either required (>20 m) or opting to use AIS [46], and thus has incomplete coverage of vessels smaller than 20 m. Hours of vessel activity were averaged per 1° grid cell within the NWA study area across all five years. A t-test was conducted to determine any significant seasonal differences in vessel activity.

### Sightings data

In addition to incidents and vessel activity, our study compiled 483,003 opportunistic sightings (presence-only) of baleen whales in the North Atlantic between 1985 and 2023 from various sources, including DFO-Maritimes opportunistic sightings database and the Whitehead Lab whale survey databases [47], the North Atlantic Right Whale Consortium [48], Environment Canada Seabirds at Sea (ECSAS) [49], the Réseau D’observation de Mammifères Marins (ROMM) [50,51], and the Ocean Biodiversity Information System (OBIS) [52]. These observations (where the species identification was reliable and quality checked for all sources except OBIS where this was not possible) were used to create the species distribution models described below. Although some data were available prior to 1985 (i.e. from 1904 onwards), we only retained records from 1985 (93% of data) as this timeframe aligns with the environmental data used in the SDMs, thus ensuring consistency between whale and environmental observations. As most whale observations were collected in warmer months when observer effort and whale presence in the region is highest as a result of baleen whale migration [40], only sightings recorded between April 1st and October 31st of each year were used; thus, the SDMs reflect spring, summer, and early fall occurrence of baleen whales in the region. Most whale sightings were made from vessels, potentially leading to a correlation between the vessel activity data and the sightings; hence the rationale for conducting the analysis using species distribution models rather than sightings, to identify areas of high potential suitability in regions with low vessel traffic and minimize any confounding effect. Furthermore, effort and absence data associated with the sightings was either not recorded or not made available for use in this study.

All sightings were aggregated to a 10x10 km grid across the entire North Atlantic Ocean to match the environmental data and allow for high-resolution species distribution model projections, described in more detail below, using R Version 4.2.1 [53]. Each grid cell with one or more whale sightings was assigned a presence value of 1, thus limiting any potential bias of cells containing numerous records through a spatial filtering process [54].

### Environmental data

Environmental data used to construct the SDM were sourced from high-resolution Community Earth System Model (CESM) simulations from CMIP6 [55,56]. We used recent hindcasts (1985–2015) and projections for 2035–2045 and 2045–2055, both based on the 2xCO_2_ climate scenario under which emissions are expected to double by 2100 (roughly similar to the high-emissions RCP8.5 pathway) [56]. This scenario and associated hindcasted/projected time periods are often used in modelling studies of species under climate change [57–60]. Data were extracted at a 10km resolution. Bathymetric data at the same resolution were obtained from the General Bathymetric Chart of the Oceans (GEBCO) [61]. All environmental data were aligned to the same North Atlantic Ocean 10km grid as the opportunistic sightings using the *sf* package in R [62]. We removed highly correlated (> 0.7) environmental variables (such as euphotic layer depth) and those deemed non-relevant to baleen whale habitat (such as abyssal zones) [63]. The remaining variables encompassed key oceanographic and biological factors hypothesized to affect baleen whale food availability and habitat [42,43,64]: sea surface temperature (SST) and sea-surface salinity (SSS) relate to the physiological suitability of habitat, nutrient availability and prey densities [65,66]; net primary production (NPP) affects potential food supply [67], as increased primary productivity provides food for lower trophic level consumers such as copepods and other zooplankton, the primary diet for many baleen whales [11]; bathymetric features such as ocean depth, slope, and shelf presence shape nutrient cycling patterns [68–70], affecting the availability of primary consumers and influencing baleen whale habitat selection [11]. Given the centrality of prey availability and physiological suitability in habitats for all baleen whales [40], these variables were uniformly applied for all six species.

### Species distribution models

To reduce the impacts of any potential observational effort bias in the species presence-only data we used species distribution modelling to generate habitat suitability maps, which can project areas of high habitat suitability in regions that are undersampled [42,71–73]. This approach is a popular way to investigate cetacean distributions when there is limited information on sampling effort [74–76]. We employed a weighted multi-model ensemble approach using the *Biomod2* package in R [77], according to SDM practices described by Robinson et al. [78]. The default *Biomod2* parameters were used, unless otherwise indicated. Our ensemble approach averaged across three statistical models for each whale species: a generalized linear model (GLM), a random forest model (RF), and a maximum entropy (MaxEnt) model. These models were specifically selected for their effectiveness with presence-only and zero-inflated sightings data and generally good predictive power [77–81]. Due to the lack of verified ’absence’ records, 10,000 pseudo-absences were randomly generated for each species [80,82,83]. Each model’s performance was evaluated through cross-validation, partitioning the data into 80% training and 20% testing sets [80]. This process was repeated five times, and performance was quantified using True Skill Statistic (TSS) scores [84,85], as Area Under the Receiver-Operator-Curve (AUC) values have been shown to be less accurate for evaluating model accuracy when data are highly biased data [86,87]. Only models with a mean TSS above 0.7 were included in the final ensemble for each species, with the contribution of each model weighted by its mean TSS score [80]. The relative contribution of environmental variables to each model was evaluated using a Mean Decrease Accuracy (MDA) approach [80].

As our study included future projections, it is entirely possible that parts of the region of interest would experience environmental conditions beyond those of the present day, and hence fitting SDMs to just the region of interest would underestimate species niches and hence overestimate climate impacts [80]. To account for this, we fit SDMs to data for the entire North Atlantic Ocean, to better characterize the environmental niche of each species, and to limit ’clamping’, whereby model projections become unreliable due to environmental variables extending beyond their training range [80,88]. We then restricted and retained model output for interpretation to just the Northwest Atlantic region of interest, for which habitat suitability values (HSVs) were extracted for both current and future conditions under the 2xCO_2_ climate scenario. For the analysis of indicent and vessel overlap described below, outputs were aggregated to the same 1°x1° grid (using mean ensemble HSV per grid cell) as the incident and vessel activity data, to be used in the analyses of incidents and vessel overlap described below.

### Independent model assessment

An independent assessment of the models’ predictive performance was conducted by comparing model habitat suitability projections to an independent data set of 41,371 acoustic detections of blue, fin, and humpback whales (presence/absence) provided by JASCO and Fisheries and Oceans Canada [47], and 4,639 acoustic detections of sei whales (number of detections or presences) also provided by Fisheries and Oceans Canada [89]. Minke and NA right whale acoustic data were not available. For blue, fin, and humpback whales, presence or absence was recorded by 25 acoustic receivers throughout the study area between 2015–2017; only data between April and October were used to match the models’ seasonality. Habitat suitability at a 10km resolution from the model ensemble was calculated for each acoustic record (both detection and non-detection) from multiple acoustic receivers using QGIS. Following this, the mean habitat suitability for all presence (detections) and absence (non-detections) records across the timeframe was calculated. For the separate database of sei whale acoustic detections from 2015 to 2017, we calculated the average habitat suitability for sei whales at the locations of 10 acoustic receivers across the study area.

### Relationship between incidents, vessel activity, and whale habitat

To determine if vessel activity and likelihood of baleen whale presences (proxied by habitat suitability) were significant predictors of incidents, a generalized linear model (GLM) was applied. Before constructing the model, data exploration techniques recommended by Zuur et al. [63] were applied to ensure all model assumptions were met. Outliers were removed, and homogeneity, normality, zero-inflation, collinearity, and interactions and independence between variables were checked for each data set [63]. Present-day baleen whale habitat suitability (*HSV*, mean habitat suitability per 1° grid cell) and vessel density (*V*, mean number of vessel hours per 1° grid cell between 2017–2021) were the predictor variables included in the model, with observed baleen whale incidents (*NI*, number of baleen whale incidents per 1° grid cell) the response variable. Spatial autocorrelation was checked using a Moran’s plot of residuals and was non-significant; hence an auto-covariate term was not included. A negative-binomial (*NB*) distribution was used for the response variable as it consisted of over-dispersed, zero-inflated count data. The *pscl* package in R [90] was used to fit the model. The analysis was repeated six times, once for each of the baleen whale species. The final model for each species was therefore specified as:

$$NI_{i}∼NB(\mu_{i},\theta_{i})$$ (1)

$$log(NI_{i})=\beta_{0}+\beta_{1}\;V+\beta_{2}\;HSV_{i}$$ (2)

where for each grid cell and the *i*th species *NI*_*i*_ represent the number of incidents, *V* the vessel activity, *HSV*_*i*_ the present-day habitat suitability, and *μ* is the mean and *θ* the dispersion parameter of the negative binomial distribution.

### Relative incident risk hotspots

Relative incident risk hotspots (i.e. areas where the relative risk of a whale and a vessel encountering each other in the same grid cell is high) were calculated for each of the six species following methods developed by Vanderlaan et al. [91]. First, the normalized relative probability *W*_*i*,*j*,*k*_ of a whale of species *i* in time period *j* occupying a grid cell *k* relative to the other *n—1* grid cells in the study area was calculated as:

$$W_{i,j,k}=\frac{{HSV}_{i,j,k}}{\sum_{k=1}^{n}\;{HSV}_{i,j,k}}$$ (3)

with the assumption that *HSV* and relative probability of occupancy scale linearly. Second, the normalized relative probability *B*_*j*,*k*_ of a vessel in time period *j* occupying a grid cell *k* relative to the other *n—*1 grid cells present in the study area was calculated using a similar approach [91] for all three time-periods:

$$B_{j,k}=\frac{V_{j,k}}{\sum_{k=1}^{n}V_{j,k}}$$ (4)

The relative risk of a whale (*W*_*i*,*j*,*k*_) encountering a vessel (*B*_*j*,*k*_) and therefore of a potential incident (*E*_*i*,*j*,*k*_) in any grid cell calculated for each grid cell as [91]:

$$E_{i,j,k}=W_{i,j,k}.B_{j,k}$$ (5)

*E*_*i*,*j*,*k*_ values were then normalized to give values ranging from zero (lowest projected relative risk) to one (highest projected relative risk). It is important to note that these values should be interpreted as relative (i.e. not absolute) and region-specific risk.

### Incident risk overlap indices and correlations

To examine the spatial congruence or overlap between observed whale incidents and projected relative risk in the Northwest Atlantic, Schoener’s *D* and Warren’s *I* similarity statistics were calculated [92,93]. These indices assess the degree of spatial overlap between two variables, yielding values between zero (no overlap) and one (perfect overlap) [94]. Values were calculated at a 1° grid resolution.

Finally, we used Spearman’s correlation analysis to determine the strength of the relationship between calculated incident risk and incident reports across all non-zero grid cells [95]. To test for statistical significance, we used a randomized reshuffling method with 1,000 permutations of the vessel density data without replacement for each grid cell. Index values were calculated for each of these permutations and compared to observed values, with an observed value outside the 95% range of this distribution considered statistically significant [96]. In addition, a Poisson-distributed regression model was run to determine if predicted incident risk was a significant predictor of observed incidents. This analysis was performed only on grid cells with one or more incidents to account for the (potential) lack of observation in cells with zero incidents (i.e., an inability to separate true zeros from a lack of observer effort, particularly offshore) [96].

## Results

### Baleen whale incident reports

**Fig 2:**
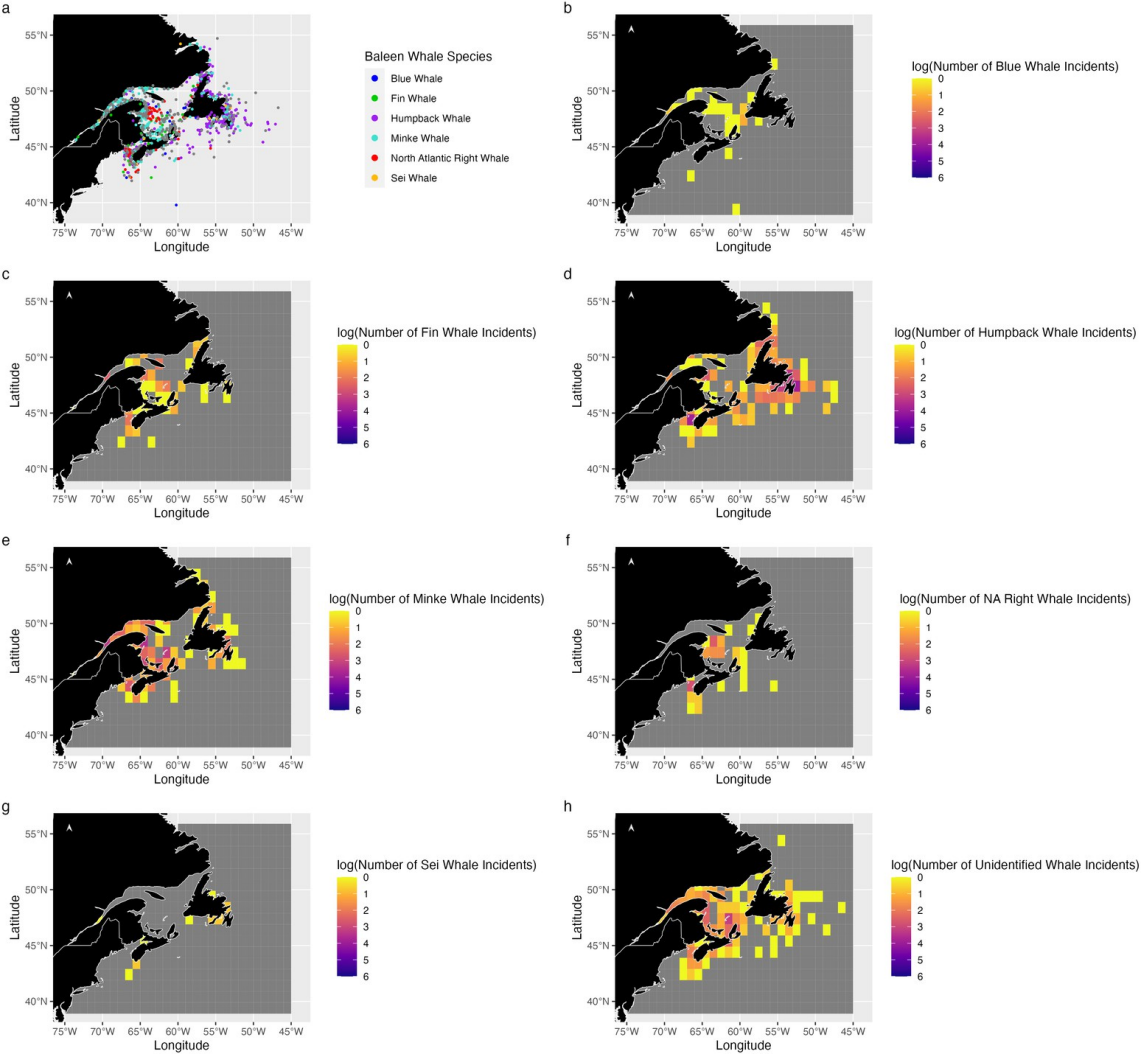
Baleen whale incidents. (a) Individual incident reports by species. Number of incidents reported per 1°x1° grid cell for (b) blue whales, (c) fin whales, (d) humpback whales, (e) minke whales, (f) NA right whales, (g) sei whales, and (h) unidentified whales. Data collected and provided by the Marine Animal Response Society, Whale Release and Strandings, and Marine Mammal Emergencies [18].

The bulk of the reported 1,110 incidents were concentrated in coastal and shelf areas, with fewer offshore (Fig 2). Notably, coastal regions like the Magdalen Islands, Bay of Fundy, Gulf of St. Lawrence, and the north-east coast of Newfoundland saw a higher number of incidents (Fig 2). 34% (457) of incidents involved humpback whales, 29% (391) involved minke whales, 7% (102) involved fin whales, 5% (68) involved NA right whales, 2% (27) involved blue whales, and 1% (14) involved sei whales.

### Vessel activity patterns

**Fig 3:**
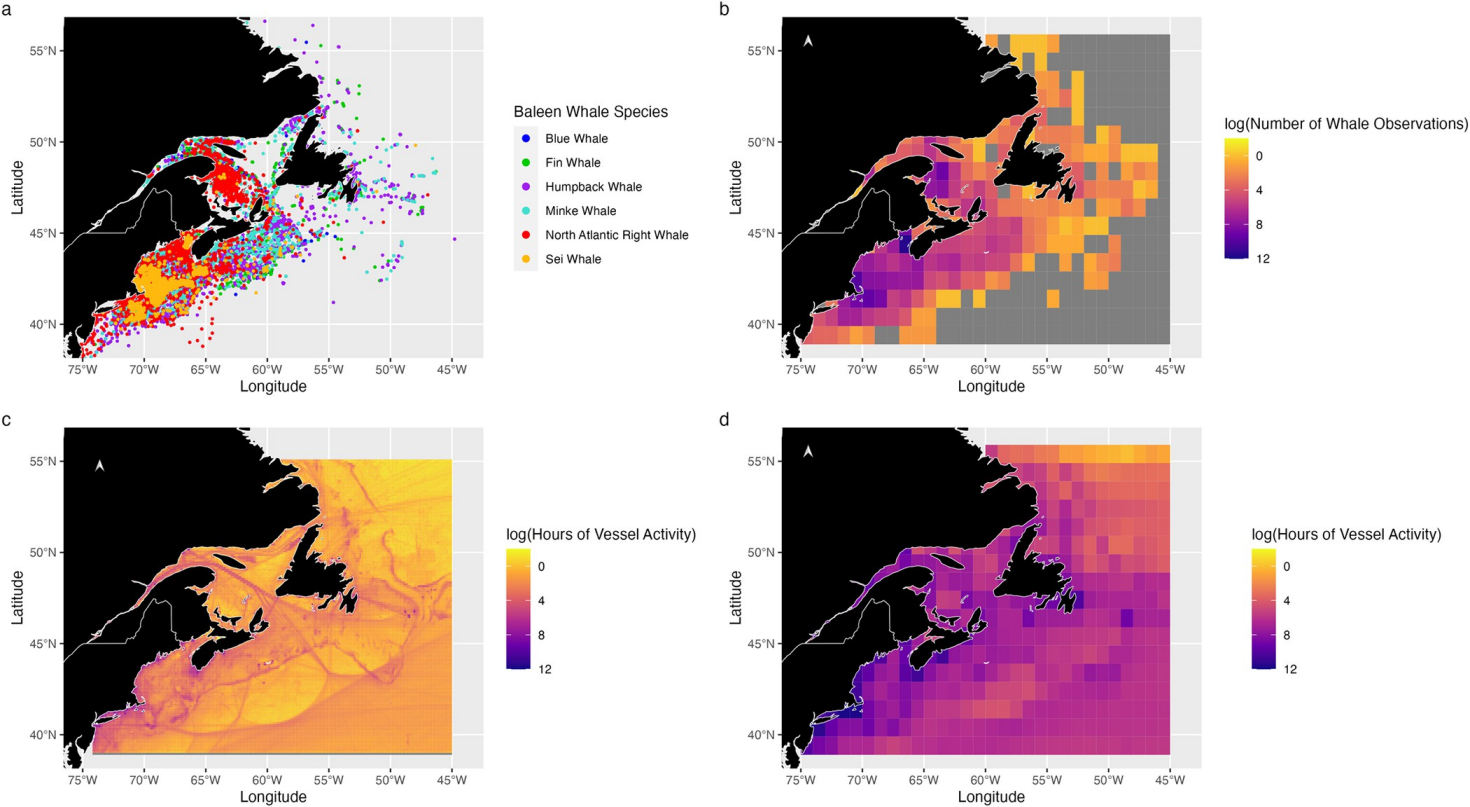
Baleen whale sightings and vessel activity. (a) Individual baleen whale sightings by species, (b) number of baleen whale aggregated by 1°x1° grid cell, (c) vessel activity (hours of activity across 2017–2021 per 0.1° x 0.1° cell), and (d) vessel activity per grid cell (hours of activity aggregated by 1°x1° cell). Data provided by DFO-Maritimes opportunistic sightings database and the Whitehead Lab, the North Atlantic Right Whale Consortium, Environment Canada Seabirds at Sea, the Réseau D’observation de Mammifères Marins, the Ocean Biodiversity Information System, and Global Fishing Watch.

From 2017 to 2021, there were 82,141,732 hours of AIS-detected vessel activity, mostly including shipping and commercial fishing, in the region, with an annual average of 16,428,346 hours (±2,077,974 SE) per year. Activity peaked during summer, with markedly fewer vessel hours in other seasons (*P*<0.001). At a 1° grid resolution, the average annual activity was 3,501 hours per cell across 2017–2021 (±528 SE) [41]. The densest vessel activity concentrations were identified around the Gulf of St. Lawrence shipping channel, the Scotian Shelf and Bay of Fundy, both of which are popular fishing areas, off Cape Breton Island’s northern coast, and transit routes near Prince Edward Island and Nova Scotia including the approaches to Halifax Harbour (Fig 3C and 3D). In contrast, activity was sparse north of Labrador (Fig 3C and 3D).

### Baleen whale sightings

Between 1985 and 2023, there were a total of 483,003 recorded sightings of baleen whales in our database. Most sightings were from ships, with fewer from the air and the shore. Humpback whales dominated with 263,261 sightings (~55%). NA right, minke, and fin whales followed with 71,772 (~15%), 67,048 (~14%), and 60,924 (~12%) sightings, respectively. Blue and sei whales were less frequently observed with 11,390 (~2%) and 8,608 (~2%) sightings, respectively. The remaining sightings can be attributed to occasions where the species could not be identified. At a 1° resolution, the Bay of Fundy, the Scotian Shelf, the Gulf of St. Lawrence, and the north-east coast of Newfoundland had most sightings, while regions far offshore or north of Newfoundland’s north-east coast had much fewer reports (Fig 3A and 3B).

### Species distribution models

All species distribution models had very high classification accuracy (TSS > 0.90) (Table 1). The RF model consistently achieved the highest accuracy (TSS > 0.93) followed by the MaxEnt and GLM models (Table 1) for all species except the blue and sei whale where the GLM was the most accurate (TSS > 0.96) followed by MaxEnt and RF. The proportionally weighted ensemble model marginally improved individual model performance (TSS > 0.93 across all species) and was used for all analyses reported here (Table 1). Environmental variables of importance were moderately consistent across species. In general, SSS emerged as the most important environmental variable, with some individual models and the humpback ensemble predicting SST as the variable with greatest importance (S2 Table). The second most important covariate was typically SST, except for humpback and blue whales. The third most important covariate varied: NPP for fin and NA right whales; depth for humpback, minke, and sei whales; and SST for blue whales. Across all species, shelf and slope ranked as the least impactful variables (S2 Table).

**Table 1:** True Skills Statistic (TSS) values for individual species distribution models. TSS values are shown for Generalized Linear, Random Forest, and MaxEnt species distribution models, and an ensemble model that is a weighted average of all three. TSS values range from zero to one, with values closer to one indicating better model performance.

Species	GLM	RF	MaxEnt	Ensemble
	TSS	TSS	TSS	TSS
Blue whale	0.977	0.947	0.912	0.986
Fin whale	0.943	0.955	0.938	0.954
Humpback whale	0.916	0.940	0.923	0.935
Minke whale	0.945	0.954	0.945	0.955
NA right whale	0.956	0.960	0.958	0.963
Sei whale	0.969	0.966	0.966	0.977

**Fig 4:**
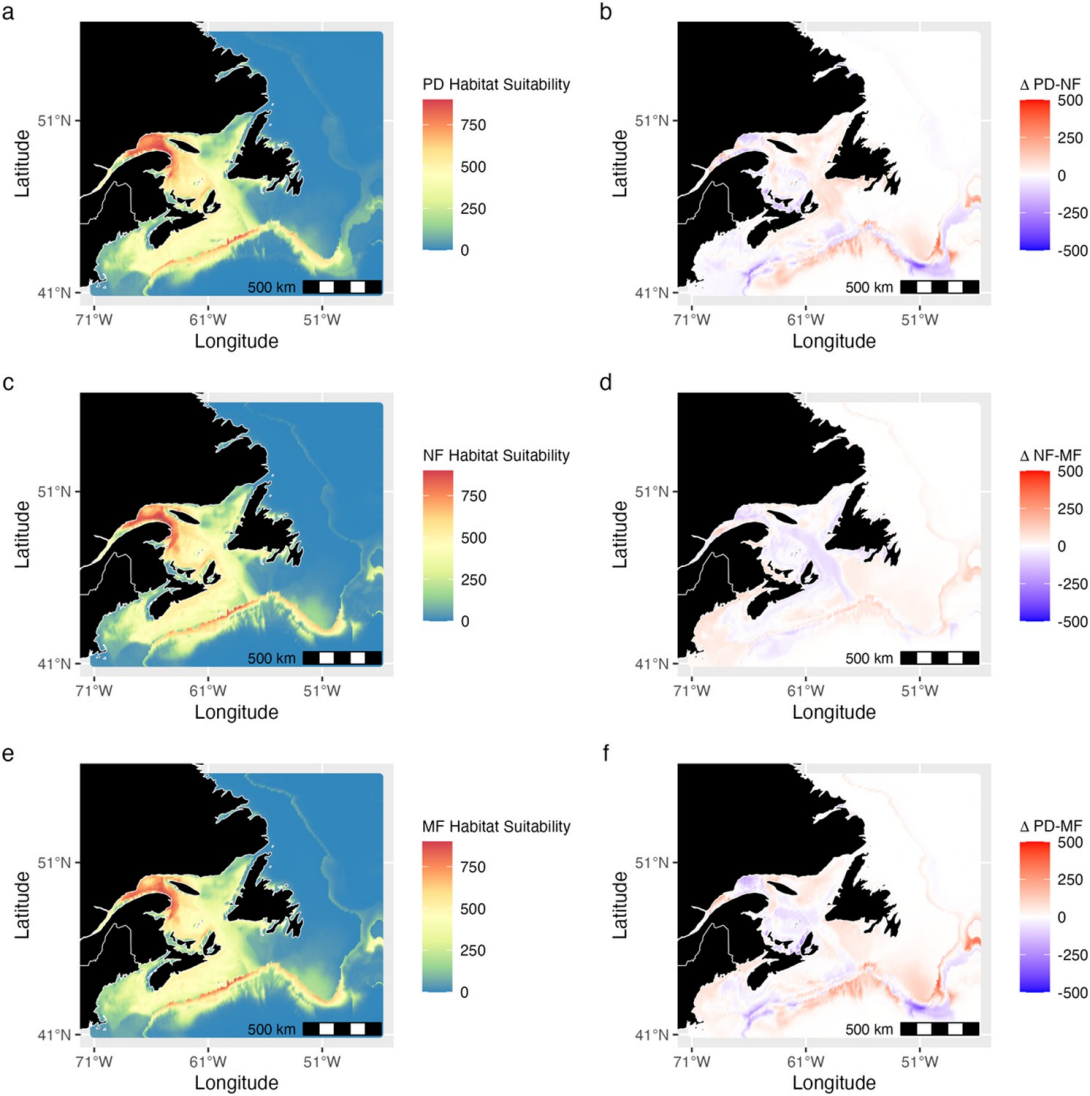
Habitat suitability estimates for blue whales. Projections from an ensemble species distribution model show (a) present-day (PD) habitat suitability (1985–2015). (b) Projected change in suitability from the present day to near-future (NF). (c) Near-future habitat suitability (2035–2045). (d) Change in habitat suitability from the near to mid-future (MF). (e) Mid-future habitat suitability (2045–2055). (f) Change in habitat suitability from the present day to the mid-future. Future projections refer to a climate scenario assuming a doubling of CO_2_ concentrations. Red colours reflect high habitat suitability values (HSV) and blue colours reflect areas with lower habitat suitability. Habitat suitability values reflect spring, summer and fall, but not winter suitability. For other species see Figs 5 and S2–S5.

**Fig 5:**
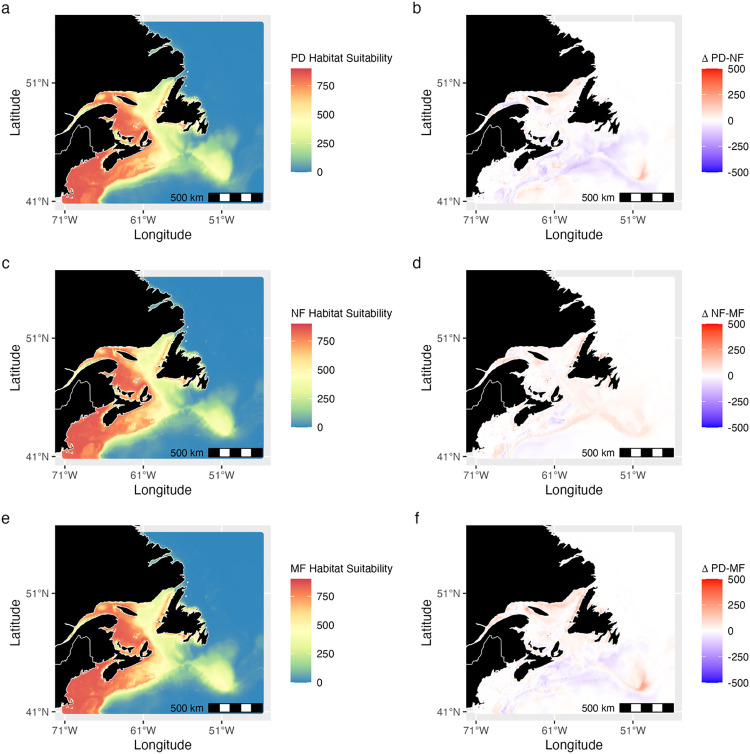
Habitat suitability estimates for NA right whales. Projections from an ensemble species distribution model show (a) present-day (PD) habitat suitability (1985–2015). (b) Projected change in suitability from the present day to near-future (NF). (c) Near-future habitat suitability (2035–2045). (d) Change in habitat suitability from the near to mid-future (MF). (e) Mid-future habitat suitability (2045–2055). (f) Change in habitat suitability from the present day to the mid-future. Future projections refer to a climate scenario assuming a doubling of CO_2_ concentrations. Red colours reflect high habitat suitability values (HSV) and blue colours reflect areas with lower habitat suitability. Habitat suitability values reflect spring, summer and fall, but not winter suitability. For other species see Figs 4 and S2–S5.

The Bay of Fundy, Scotian Shelf, Laurentian Channel, Gulf of St. Lawrence, the south-west coast of Newfoundland, and areas near the Flemish Cap were habitats with high predicted suitability across all baleen whale species (Figs 4A, 5A and S2A–S5A). Specifically, the Gulf of St. Lawrence showed high suitability for blue and NA right whales, while the Bay of Fundy and Scotian Shelf stood out for humpback, NA right, minke, and fin whales. Blue whales, however, appeared to have more limited suitable habitat, primarily along shelf edges and sharp bathymetric features (Fig 4A). Sei whale habitat suitability was unique in its high suitability projections in the southern part of the Bay of Fundy (S5A Fig). Further from the shelf, habitat suitability generally decreased for all species, except around waters near the Flemish Cap and Grand Banks (Figs 4A, 5A and S2A–S5A).

Under the 2xCO_2_ climate scenario for 2035–2045 (near future), the Gulf of St. Lawrence was projected to remain suitable for blue, fin, and sei whales but slightly less so for others (Figs 4B, 4C, 5B, 5C, S2B, S2C–S5B and S5C). Suitability in the Laurentian Channel was projected to increase for blue and fin whales. The Scotian Shelf’s suitability was projected to increase for blue and fin whales but decrease for humpback, NA right, and sei whales. Offshore regions were projected to remain less suitable for all species (Figs 4B, 4C, 5B, 5C, S2B, S2C–S5B and S5C).

When projected into the mid-future (2045–2055), the Scotian Shelf’s suitability was expected to increase for all species except blue whales (Figs 4D, 4E, 5D, 5E, S2D, S2E–S5D and S5E). The Gulf of St. Lawrence was projected to become more suitable for most species, except blue, fin, and sei whales. Changes in offshore habitat suitability were minimal, but areas near the shelf edge showed increased suitability for all species (Figs 4D, 4E, 5D, 5E, S2D, S2E–S5D and S5E).

Comparing mid-future (2045–2055) projections to the present-day, the Laurentian Channel was projected to see increased suitability for all but the humpback and minke whales (Figs 4F, 5F and S2F–S5F). The Gulf of St. Lawrence was projected to become more suitable for half of the species, excluding the humpback, minke, and sei whales. The Scotian Shelf, however, was likely to become less suitable for humpback, minke, NA right, and sei whales (Figs 4F, 5F and S5F). Offshore regions, especially near the Flemish Cap and Grand Banks, were projected to see an increase in suitability for all species (Figs 4F, 5F and S2F–S5F).

### Independent model assessment

For blue, fin, and humpback whales, areas with acoustically detected presences consistently showed higher average habitat suitability compared to areas of non-detection (S3 Table). Regarding sei whales, the locations with the highest counts of definite sei whale detections also had the highest habitat suitability values in comparison to areas with lower detection frequencies (S3 Table).

### Relationship between incidents, vessel activity, and whale habitat

Both vessel activity and habitat suitability were found to be significant predictors of blue whale incidents (Table 2). For humpback whales, vessel activity was the only variable found to be a significant predictor of incidents (Table 2). For minke and NA right whales, only habitat suitability was found to be a significant predictor of incidents (Table 2). Neither habitat suitability nor vessel activity had a significant relationship with the observed number of incidents for fin or sei whales (Table 2). In summary, vessel activity was a significant predictor of incidents for 2 species, and habitat suitability a significant predictor for 3 species (Table 2).

**Table 2:** Predicting baleen whale incidents from habitat suitability and vessel density. Estimated regression parameters, standard errors, and p-values for the zero-inflated negative-binomially distributed generalized linear model used to predict baleen whale incidents. Values are reported for each individual species model.

Species	Covariate	Estimate	Standard Error	p-Value
Blue whale	Vessel Hours	<0.001	<0.001	<0.001*
	Habitat Suitability	<0.001	<0.001	<0.001*
Fin whale	Vessel Hours	<0.001	<0.001	0.805
	Habitat Suitability	0.002	0.001	0.313
Humpback whale	Vessel Hours	<0.001	<0.001	0.012*
	Habitat Suitability	<0.001	<0.001	0.739
Minke whale	Vessel Hours	<0.001	<0.001	0.770
	Habitat Suitability	<0.001	<0.001	0.004*
NA right whale	Vessel Hours	-0.096	0.208	0.640
	Habitat Suitability	0.010	0.002	<0.001*
Sei whale	Vessel Hours	0.762	0.500	0.127
	Habitat Suitability	-0.002	0.003	0.513

### Projected relative incident risk hotspots

**Fig 6:**
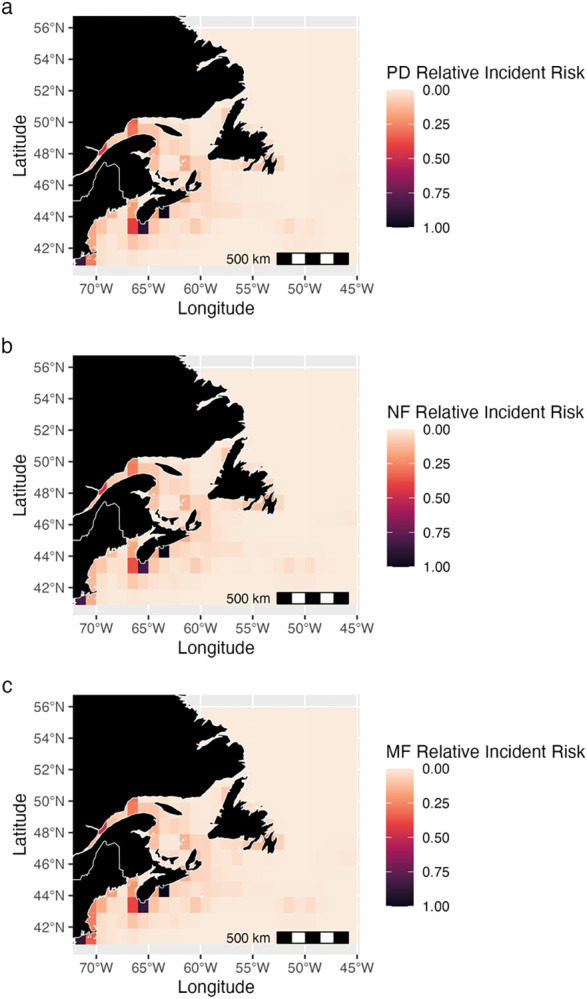
Changes in relative incident risk for blue whales. Relative incident risk (a) for the present day (PD) (1985–2015) for all vessels, (b) for the near-future (NF) (2035–2045), and (c) mid-future (MF) (2045–2055) under climate scenario 2x CO_2_. Darker colors indicate areas where blue whales are predicted to be more vulnerable to incidents based on species and vessel distribution. Values across the mapped area are normalized to sum to one, and hence are relative values and cannot be compared in absolute terms between species, only in terms of spatial patterns.

**Fig 7:**
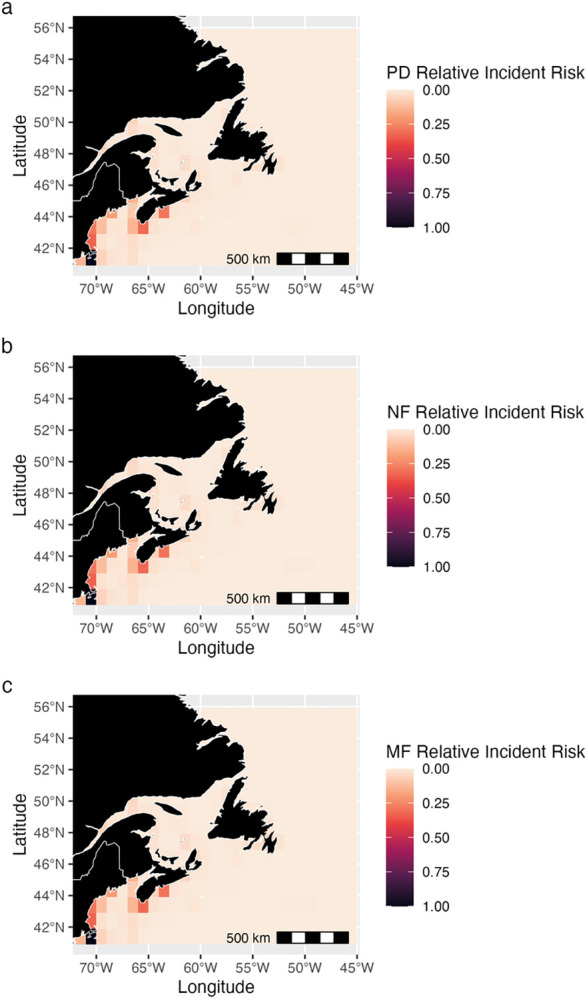
Changes in relative incident risk for NA right whales. Relative incident risk (a) for the present day (PD) (1985–2015) for all vessels, (b) for the near-future (NF) (2035–2045), and (c) mid-future (MF) (2045–2055) under climate scenario 2x CO_2_. Darker colors indicate areas where NA right whales are predicted to be more vulnerable to incidents based on species and vessel distribution. Values across the mapped area are normalized to sum to one, and hence are relative values and cannot be compared in absolute terms between species, only in terms of spatial patterns.

Coastal and shelf areas throughout the entire study region, especially within the Bay of Fundy, Gulf of St. Lawrence, the Laurentian Channel, and waters off St. John’s, Newfoundland and Labrador and Halifax and Yarmouth, Nova Scotia, were identified as areas of high relative incident risk (Figs 6A, 7A and S6A–S9A). There was also projected to be an area of high relative incident risk near the Flemish Cap, east of Newfoundland (Figs 6A, 7A and S6A–S9A). Areas where relative incident risk was projected to be high did not differ substantially from present-day conditions in the near-future (2035–2045) climate for all species. However, slight changes in relative incident risk in some areas were projected (Figs 6B, 7B and S6B–S9B). Changes in relative incident risk from the present day to the mid-future were also limited (Figs 6C, 7C and S6C–S9C). The main areas with high relative incident risk up to mid-century were around the ports of Yarmouth, Halifax, St. John’s, as well as around the Flemish Cap (Figs 6, 7 and S6–S9).

### Incident risk overlap indices and correlations

**Fig 8:**
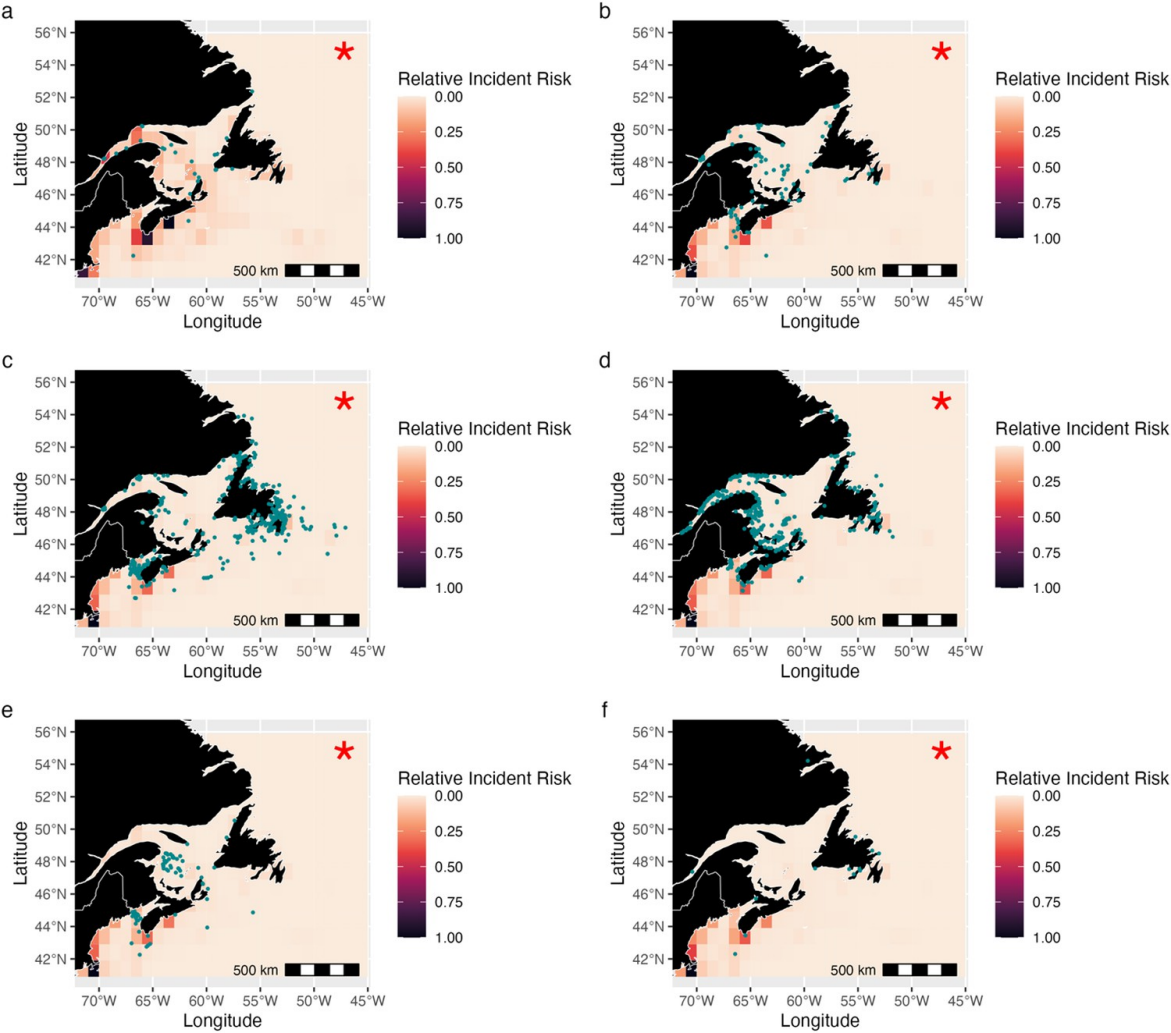
Relative incident risk and incident reports. Present-day relative incident risk for the (a) blue, (b) fin, (c) humpback, (d) minke, (e) North Atlantic right, and (f) sei whale. Incidents from between 2004 and 2019 for each baleen whale have been overlaid using teal dots. Data collected and provided by the Marine Animal Response Society, Whale Release and Strandings, and Marine Mammal Emergencies [18]. Red star indicates a significant overlap between areas of high incident risk and incident reports. Values across the mapped area are normalized to sum to one, and hence are relative values and cannot be compared in absolute terms between species, only in terms of spatial patterns.

Overlap indices were calculated to determine the overlap between present-day projected relative incident risk and actual incident reports (Table 3). All whales showed significant overlap between incident risk and incident reports, as evidenced by a positive Spearman’s correlation (Fig 8). Humpback and minke whales showed the strongest correlation (Table 3).

**Table 3:** Overlap between present-day relative incident risk and incident reports. Schoener’s D, Warren’s Index, and Spearman’s Correlation for relative incident risk and baleen whale incident reports for the present day. Asterisk indicates the reported value was significant at the 0.05 level (the value was outside of the 95% confidence interval bound generated from the simulations—see Methods). Values are reported for each individual species.

Species	Schoener’s D	Warren’s Index	Spearman’s Correlation
Blue whale	0.21*	0.39*	0.32*
Fin whale	0.21*	0.41*	0.36*
Humpback whale	0.25*	0.50*	0.47*
Minke whale	0.27*	0.50*	0.50*
NA right whale	0.15*	0.32*	0.33*
Sei whale	0.11*	0.24*	0.18*

When the number of reported incidents (or incident report effort) was modelled as a function of the projected relative risk of an incident, the projected relative risk was not a significant predictor of the number of incidents for individual species, except for humpback and minke whales (P<0.05) (Table 4). However, the average relative incident risk for all baleen whales combined was a significant predictor of the observed number of baleen whale incidents (P<0.001) (Table 4). Nonetheless, there was limited explanatory power as most models explained less than 10% of the spatial variance in observed incident reports, indicating a need for caution when interpreting number of incidents (Table 4). In summary, the differences between overlap indices and the regression suggests that projected incident risk at least in part captures locations where incidents are more likely to occur but has limited ability to predict the number of incidents occurring in those locations.

**Table 4:** Predicting number of baleen whale incidents. Estimated regression parameters, standard errors, p-values, and explained variances (R^2^) for the generalized linear model used to predict baleen whale incidents as a function of relative incident risk. Values are reported for each individual species model and for all baleen whale species combined.

Species	Estimate	Standard Error	p-Value	Variance Explained (R^2^)
All baleen whales	4.896	0.391	<0.001*	0.080
Blue whale	0.470	1.643	0.775	0.023
Fin whale	0.899	0.116	0.142	0.027
Humpback whale	<0.001	<0.001	<0.001*	0.034
Minke whale	2.405	0.642	<0.001*	0.029
NA right whale	0.271	1.670	0.871	<0.001
Sei whale	1.276	2.217	0.565	0.179

## Discussion

We analyzed baleen whale habitat suitability and incident risks in comparison to present-day vessel activity, employing species distribution models to mitigate the effect of spatially uneven observation effort [40,42]. We projected specific regions as high-risk for whale-vessel incidents for the six baleen whale species in the region both in the present day and under climate change projections up to the year 2055.

### Species distribution model performance and variables of importance

The habitat suitability models in this study performed well, with TSS values above 0.90 across all species [80]. Such high model performance values are not uncommon [97,98]. The weighted ensemble models outperformed individual approaches, as expected. The comparison with independently-derived acoustic data (see below) enabled a separate assessment of model performance.

All species except humpback whales favored areas with lower salinity for habitat suitability, predicting key whale habitat in the region, such as the GSL (Figs 4A, 5A, S2A–S5A and S11). This result aligns with studies suggesting that estuarine, coastal and shelf areas with lower salinity tend to exhibit higher productivity, potentially enhancing prey availability for baleen whales [99–101]. However, some studies suggest preference for higher salinity, primarily based on Pacific populations [65,102]; here, SDMs without the SSS covariate had substantially lower model accuracy (TSS < 0.8), suggesting that it also plays a role in determining important habitat suitability in this region.

SST emerged as the second-most influential variable, reflecting the impact of temperature on whale physiology and prey availability [65,66,76]. Cooler waters were found to be more suitable, possibly because they tend to be more nutrient-rich due to increased mixing, supporting higher productivity and prey availability [103] (S12 Fig). This aligns with observed shifts in NA right whale prey, *Calanus finmarchicus*, to cooler northern waters, and therefore the whales themselves, which have been attributed to climatic changes [11,104]. Similar findings were reported in other studies which modelled baleen whale habitat suitability on the Scotian shelf, eastern North Atlantic, and Southern right and humpback whales in South African waters [40,76,105,106].

NPP and bathymetric depth emerged as the third and fourth most influential variables, depending on the species. While higher NPP corresponded to higher habitat suitability, limited variability in mean observed NPP through space within the model region likely tempered the model’s ability to detect a strong NPP-whale presence relationship (S13 Fig). Nonetheless, the relationship supports findings from similar studies linking baleen whale habitat to areas of high productivity [11,40,107]. There may be a lag time between phytoplankton blooms and regional secondary productivity (e.g. an emergence of *Calanus* from diapause or zooplankton) that is not accounted for in these models that weakens the relationship between primary productivity, secondary productivity, and suitable habitat [76,108,109]. Additionally, not all baleen whales occupy the same trophic level, and so using NPP as a proxy may affect accuracy or model performance in different ways for difference species.

Bathymetry (i.e. ocean depth) influenced habitat suitability for the open-ocean species blue and fin whales (S14 Fig). Geological features can enhance nutrient mixing and productivity, aligning with findings from other baleen whale habitat studies on the Scotian Shelf, Newfoundland waters, and in South African waters [40,69,76,106,110]. While these environmental variables may also serve as proxies for prey availability and habitat preference, incorporating actual prey data such as zooplankton concentration, could enhance the model’s biological accuracy in projecting baleen whale habitat suitability–and potentially improve future projections [105,111,112]; however, such projections were not available for use in our study.

### Habitat suitability

The species distribution models consistently indicated high habitat suitability in coastal and shelf areas across different time periods (Figs 4, 5 and S2–S5). Fin, minke, and humpback whales displayed slightly greater offshore suitability near the Scotian Shelf, Flemish Cap, and Grand Banks (Figs 4 and S2). This aligns with existing research that underscores the significance of these coastal and shelf regions as preferred feeding grounds for all species, particularly blue, fin, humpback, and NA right whales [27,76,113,114]. These areas are known for abundant prey resources such as small fish, krill, copepods, and other zooplankton. For instance, the Bay of Fundy (including the Roseway and Grand Manan Basins), the Scotian Shelf, Gulf of St. Lawrence, and Grand Banks are all recognized as important baleen whale feeding grounds due to the availability of vital prey species [26,27,113–116]. In our analysis, the Gulf of St. Lawrence and St. Lawrence Estuary emerged as a region of habitat suitability, especially for the NA right whale (Fig 5). This corresponds with a well-documented shift in prey distribution, with *Calanus finmarchicus* moving from the Bay of Fundy to the Gulf of St. Lawrence (Record et al. 2019). Similarly, blue whales, known to frequent this area, exhibited elevated habitat suitability (Fig 4), mirroring their observed presence [26]. Blue, humpback, fin, and minke whales were also projected to have high habitat suitability in these two regions (S2–S4 Figs), likely primarily due to abundant prey resources. Sei whales showed a preference for the Bay of Fundy and the Scotian Shelf, aligning with acoustic research that confirms their presence in these prey-rich regions [27]. Conversely, the models projected suitable habitat north of Newfoundland and along the Labrador coast for fin, humpback, and minke whales (S2–S4 Figs). While these areas have fewer acoustic detections [27], they may represent crucial, yet less-explored, baleen whale habitats. One point to note is that our model was created using spring, summer, and fall presences, when the whales are likely frequenting near-shore and shelf feeding grounds in the region. As these species are migratory [116]; enhanced offshore detection efforts and a model incorporating migration pathways might reveal higher habitat suitability in deeper offshore waters when transiting to their winter habitats.

Using the CESM ESM with a 2xCO_2_ scenario [55], future projections indicated similar baleen whale habitat suitability for the near and mid-future (Figs 4, 5 and S2–S5). This is relatively unsurprising, as many of the impacts of climate change on the marine environment are likely to play out in the second half of this century, with low- and high-emission scenarios aligning until then [117]. However, there are projected to be localized increases in suitability for some species, notably in the Gulf of St. Lawrence and Scotian Shelf (Figs 4, 5 and S2–S5), driven by projected changes in temperature, salinity, and primary production, as reflected in the SDM. Offshore regions may also become more suitable due to projected local cooling in some areas. In the mid-future, slight suitability shifts occur, particularly around the Scotian Shelf and Gulf of St. Lawrence. NA right whales show heightened suitability near Newfoundland’s southwest coast, while other species show increased suitability offshore (Figs 4, 5 and S2–S5). Comparable studies on future baleen whale distributions under climate scenarios are relatively scarce [118]. Similar projected future range contractions for cetaceans in areas with high prey density were identified in the eastern North Atlantic by Lambert et al. [105], in waters surrounding New Zealand by Peters et al. [76], and in arctic regions by Chambault et al. [119]. Global Aquamaps projections differ markedly [120], potentially due to regional dataset availability limitations and a large-scale focus. While our models reveal local shifts, they don’t capture the broader poleward or offshore shifts suggested in some global studies [31,39,121–123].

Building on the findings detailed earlier, our models predict observable shifts in habitat suitability over several decades, with particular emphasis on NA right, humpback, and blue whales [11,29,124–126]. Importantly, the congruence between areas of higher projected habitat suitability and regions with notable acoustic detections for species like blue, fin, humpback, and sei whales not only reinforces the predictive reliability of our habitat suitability models but also highlights their value in identifying specific habitat preferences for these species. Our models projected consistently higher habitat suitability when independently validated with acoustic detection data (S3 Table). Such findings underline the crucial need to preserve key feeding and migratory corridors, potentially through coupling marine protected areas with dynamic conservation strategies [127] such as seasonal management areas.

### Relationship between incidents, vessel activity, and whale habitat

At the 1° resolution, vessel activity and habitat suitability were not found to be significant predictors of incidents for fin and sei whales (Table 2). This result is contrary to existing literature suggesting fin whales are at highest risk of being involved in vessel strikes [128]. This may result from the limited coverage of small vessels in our data, or potentially reflect issues with lags between incident occurrence and detection that confound the relationship. However, blue and humpback whale incidents were significantly associated with vessel activity (Table 2), suggesting increased incident risk with vessel activity for these species, and aligning with studies that suggest greater vulnerability than for other species to vessel strikes for humpbacks and for blue whales [128,129] given their endangered status. It was interesting that vessel activity was not strongly associated with NA right whale incidents, as vessel strikes are confirmed as a prominent cause of death to this species [10,18]. This failure to predict observed incidents could be due to the very localized aggregation seen in this species and others, the fact that these whales may drift for a while before being reported, only a subset of vessels are captured by AIS data, or other factors.

Projected habitat suitability predicted incidents for blue, minke, and NA right whales (Table 1). This finding emphasizes the importance of monitoring projected high-suitability areas, particularly for minke whales, which had the second-highest number of incidents among our study species. Refining habitat suitability models, particularly through enhanced observer effort in under-represented regions, and improving the documentation and investigation of incidents by aggregating more data and incorporating additional methods like tracking and acoustic studies, may help to better delineate the connections between risks and outcomes, especially for those species where current models have failed to establish clear relationships.

### Incident risk hotspots

The use of SDM-generated habitat suitability as an indicator of or proxy for baleen whale distributions in vessel strike research is gaining popularity [130,131]. Recent research has suggested combining high-resolution whale habitat suitability with vessel data to improve ship strike risk estimates, in a similar approach to that taken here [131]. Present-day projected relative incident risk hotspots align with areas of high human population density and fishing activity in Atlantic Canada [132–134]. The Bay of Fundy, an area with a high activity (Fig 3C and 3D), has been previously identified as an area of vessel strike risk for NA right whales [10,91]. Other global studies of incident risk also found high vulnerability in areas with high vessel density and activity [74,94,135]. Projected relative risk hotspot locations remain relatively stable across time, likely due to minor changes in habitat suitability (Figs 6, 7 and S6–S9) and our assumption of stable vessel activity patterns [94]. Multi-species hotspots may indicate high-risk areas where robust mitigation strategies are needed to protect several baleen whale species simultaneously.

Hotspots of predicted relative incident risk aligned spatially with reported incidents (Fig 8), though some of this signal could be due to a positive incident reporting bias near densely populated and accessible coastlines [136]. Furthermore, projected relative incident risk showed significant spatial overlap with reported incidents, again suggesting that the approach can identify potential locations of spatial risk. However, we caution against over-interpretation or over-reliance on this, as relative incident risk was generally not a significant predictor of the number of incidents for individual species (except humpback and minke whales), and the explanatory power was very low. This suggests that further quantitative data and assessment are needed before this approach can be applied in an operational manner. It particularly underscores the need for more species-specific incident data, at-sea observations and reports of incidents, and vessel activity data for smaller vessels.

### Limitations

Our results may be affected by potential sampling biases in the whale sightings and incident report data [73,87]. This is primarily due to the absence or unavailibility of associated sampling effort data, and the higher observation effort along coastlines. Additionally, most sightings were made aboard vessels, indicating the potential for a correlation between vessel activity and the sightings data. To mitigate biases in the sightings data, habitat suitability outputs from a high-resolution regional species distribution model were employed as proxies for potential whale presence, and to mitigate the issues with direct observations [40]. However, it is possible that the model’s reliance on coastal and shelf opportunistic sightings (and the use of data from spring and summer months due to increased observer effort) may have led to underestimations of habitat suitability further offshore. Addressing this bias would require increased offshore sightings effort and the incorporation of winter months into the model, when these species may be migrating. However, the present study aggregated data from multiple sources to examine regional baleen whale distributions and relative incident risk, and so provides an assessment of our current level of understanding on this issue [44,87,137]. Incorporating additional data sources such currently restricted survey data could enhance our knowledge of baleen whale habitat suitability. When comparing the species distribution models to independent acoustic detections, it is important to note that the detections used here represent a conservative estimate of whale occurrence, as the detectors cannot usually provide the number of animals present and there are several scenarios in which whales may be present but undetected: they might not vocalize, their calls could be masked by ambient noise, they may produce non-target call types, or the detectors could fail to capture calls [138]. Additionally, increased noise levels at the receiver stations during summer months may inaccurately suggest lower whale presence during this period, potentially skewing our understanding of seasonal distribution [138].

Another limitation is the potential underrepresentation of incidents, as not all are observed and reported. In addition, many incidents cannot be investigated due to logistical or financial limitations and thus the definitive causes of incidents are often not known. These challenges are compounded by the aggregation of diverse incident types such as entanglements, entrapments, mortalities, live strandings, and injuries, including those caused by vessel strikes into a single dataset. This amalgamation, which also includes incidents of unknown origins, may obscure distinct causes of whale injury and mortality. Additionally, the recorded timing and locations of these incidents may not reflect their actual occurrence sites due to factors like carcass drift or the movement of injured animals, further complicating our attempts to link whale observations with vessel activity and incident occurrences.

The study also assumes that habitat suitability is a useful linear proxy for whale occurrence in calculating relative incident risk. However, this linearity may not necessarily hold [139], although implicit in habitat suitability models is the assumption that higher habitat suitability means more favorable habitat.

Finally, our future projections necessarily assume that vessel densities remain similar over time [10], as projections of future vessel pathways and densities are not available. However, this may change with changes in vessel activity and distribution, for example in response to distributional shifts of fished target species [140]. Future research could explore modelling vessel activity changes over time and consider multiple climate scenarios over a longer time-scale [57,141] for a more comprehensive analysis of climate change impacts on baleen whale habitat suitability and incident risk.

Due to existing limitations, such as incomplete data from smaller vessels, absent effort data, and biases in incident reporting, our study likely did not identify all possible relationships between the extant whale species and vessel activity or habitat suitability. This should not be construed as evidence of non-impact, and underscores the importance of collecting more comprehensive incident data that can be used to disentangle these relationships more accurately [137,142].

### Management implications

Our study suggests a vulnerability of all baleen whale species in the NWA to harmful incidents due to the significant overlap between areas of high baleen whale habitat suitability and vessel activity, with projected relative incident risk hotspots concentrated near densely populated regions of the NWA, and such hotspots likely to remain similarly located over the coming several decades despite climate change (Figs 4, 5 and S2–S5). Such vulnerability particularly applies to SARA-listed species due to their low regional population sizes. However, protecting all whale species, even those with larger populations, remains important due to their ecological significance and vulnerability to harmful incidents [18], and the fact that we identified cross-species hotspots of risk.

Current incident management strategies that primarily target the NA right whale [35,36] likely leave other species under-protected despite being listed as endangered or of special concern. To address this, additional dynamic protection measures could adapt to changing patterns of whale distribution and human activity, ideally using multi-species approaches to minimize costs. One approach could involve seasonal management areas with speed restrictions and, if possible, vessel density control in cross-species high-suitability whale habitats or areas of projected high potential incident risk [143], or at least enhanced monitoring of these regions. These could include areas near Halifax, Yarmouth, the inner Gulf of St. Lawrence, St. John’s, and the Flemish Cap. Stricter speed and vessel regulations triggered by new whale sightings or acoustic detections of any large whale species could further enhance protection, decreasing the risk of collisions and consequent injuries to these marine animals. Additional measures like onboard observers, real-time warning systems, and improved engagement of fishing and shipping industries could help further minimize incident risk across species [143,144]. Mitigation efforts must consider all baleen whale species to be truly effective for species recovery [145]. Improving knowledge of baleen whale distribution, habitat use, and their interactions with human activities is crucial. Our study may serve as a baseline for evaluating the potential for negative human-whale interactions within the region, projected relative incident risk, and how patterns may evolve under climate change, with the aim of providing insight into cross-species baleen whale conservation and bridging existing knowledge gaps. Ensuring effective spatial management of human activity is vital for ensuring the persistence and recovery of baleen whales in an increasingly industrialized ocean.

## Supporting information

S1 Table COSEWIC and SARA Status and Population Estimates of Northwest Atlantic Large Baleen Whales. Population estimates, Committee on the Status of Endangered Wildlife in Canada (COSEWIC) and Species at Risk Act (SARA) status, and year of designation of large baleen whale populations in the Northwest Atlantic (COSEWIC 2002, 2003, 2006, 2013, 2019a,b).
(DOCX)

S2 Table Environmental variable ranking. Environmental variables of importance ranked by mean decrease accuracy (MDA) from the ensemble species distribution models for each species of baleen whale. 1 = variable of most importance, 6 = variable of least importance. SST refers to sea surface temperature, SSS refers to sea surface salinity, NPP refers to net primary productivity, and Bathy refers to bathymetry.
(DOCX)

S3 Table Comparative Analysis of Habitat Suitability in Relation to Acoustic Whale Detections. This table delineates the average habitat suitability values across different species: Blue, fin, and humpback whales, compared between areas of presence and absence, and for sei whales, compared across locations with varying frequencies of detections (only a high-detection and low-detection example has been provided).
(DOCX)

S1 Fig Whale sightings by species. Shown are reported sightings for the (a) blue whale, (b) fin whale, (c) humpback whale, (d) minke whale, (e) North Atlantic right whale, and (f) sei whale. Data provided by DFO-Maritimes opportunistic sightings database and the Whitehead Lab, the North Atlantic Right Whale Consortium, Environment Canada Seabirds at Sea, the Réseau D’observation de Mammifères Marins, and the Ocean Biodiversity Information System.
(TIF)

S2 Fig Habitat suitability estimates for fin whales. Projections from an ensemble species distribution model show (a) present-day (PD) habitat suitability (1985–2015). (b) Projected change in suitability from the present day to near-future (NF). (c) Near-future habitat suitability (2035–2045). (d) Change in habitat suitability from the near to mid-future (MF). (e) Mid-future habitat suitability (2045–2055). (f) Change in habitat suitability from the present day to the mid-future. Future projections refer to a climate scenario assuming a doubling of CO_2_ concentrations. Red colours reflect high habitat suitability values (HSV) and blue colours reflect areas with lower habitat suitability. Habitat suitability values reflect spring, summer and fall, but not winter suitability. For other species see Figs 4,5 and S3–S5 Figs.
(TIF)

S3 Fig Habitat suitability estimates for humpback whales. Projections from an ensemble species distribution model show a) present-day (PD) habitat suitability (1985–2015). (b) Projected change in suitability from the present day to near-future (NF). (c) Near-future habitat suitability (2035–2045). (d) Change in habitat suitability from the near to mid-future (MF). (e) Mid-future habitat suitability (2045–2055). (f) Change in habitat suitability from the present day to the mid-future. Future projections refer to a climate scenario assuming a doubling of CO_2_ concentrations. Red colours reflect high habitat suitability values (HSV) and blue colours reflect areas with lower habitat suitability. Habitat suitability values reflect spring, summer and fall, but not winter suitability. For other species see Figs 4,5 and S2–S5.
(TIF)

S4 Fig Habitat suitability estimates for minke whales. Projections from an ensemble species distribution model show a) present-day (PD) habitat suitability (1985–2015). (b) Projected change in suitability from the present day to near-future (NF). (c) Near-future habitat suitability (2035–2045). (d) Change in habitat suitability from the near to mid-future (MF). (e) Mid-future habitat suitability (2045–2055). (f) Change in habitat suitability from the present day to the mid-future. Future projections refer to a climate scenario assuming a doubling of CO_2_ concentrations. Red colours reflect high habitat suitability values (HSV) and blue colours reflect areas with lower habitat suitability. Habitat suitability values reflect spring, summer and fall, but not winter suitability. For other species see Figs 4,5 and S2–S5.
(TIF)

S5 Fig Habitat suitability estimates for sei whales. Projections from an ensemble species distribution model show (a) present-day (PD) habitat suitability (1985–2015). (b) Projected change in suitability from the present day to near-future (NF). (c) Near-future habitat suitability (2035–2045). (d) Change in habitat suitability from the near to mid-future (MF). (e) Mid-future habitat suitability (2045–2055). (f) Change in habitat suitability from the present day to the mid-future. Future projections refer to a climate scenario assuming a doubling of CO_2_ concentrations. Red colours reflect high habitat suitability values (HSV) and blue colours reflect areas with lower habitat suitability. Habitat suitability values reflect spring, summer and fall, but not winter suitability. For other species see Figs 4,5 and S2–S5.
(TIF)

S6 Fig Changes in relative incident risk for fin whales. Relative incident risk (a) for the present day (PD) (1985–2015) for all vessels, (b) for the near-future (NF) (2035–2045), and (c) mid-future (MF) (2045–2055) under climate scenario 2x CO_2_. Darker colors indicate areas where fin whales are predicted to be more vulnerable to incidents based on species and vessel distribution. Values across the mapped area are normalized to sum to one, and hence are relative values and cannot be compared in absolute terms between species, only in terms of spatial patterns. For other species see Figs 7, 6 and S7–S9 .
(TIF)

S7 Fig Changes in relative incident risk for humpback whales. Relative incident risk (a) for the present day (PD) (1985–2015) for all vessels, (b) for the near-future (NF) (2035–2045), and (c) mid-future (MF) (2045–2055) under climate scenario 2x CO_2_. Darker colors indicate areas where humpback whales are predicted to be more vulnerable to incidents based on species and vessel distribution. Values across the mapped area are normalized to sum to one, and hence are relative values and cannot be compared in absolute terms between species, only in terms of spatial patterns. For other species see Figs 7, 6, S6, S8 and S9.
(TIF)

S8 Fig Changes in relative incident risk for minke whales. Relative incident risk (a) for the present day (PD) (1985–2015) for all vessels, (b) for the near-future (NF) (2035–2045), and (c) mid-future (MF) (2045–2055) under climate scenario 2x CO_2_. Darker colors indicate areas where minke whales are predicted to be more vulnerable to incidents based on species and vessel distribution. Values across the mapped area are normalized to sum to one, and hence are relative values and cannot be compared in absolute terms between species, only in terms of spatial patterns. For other species see Figs 7,6, S6, S7 and S9.
(TIF)

S9 Fig Changes in relative incident risk for sei whales. Relative incident risk (a) for the present day (PD) (1985–2015) for all vessels, (b) for the near-future (NF) (2035–2045), and (c) mid-future (MF) (2045–2055) under climate scenario 2x CO_2_. Darker colors indicate areas where sei whales are predicted to be more vulnerable to incidents based on species and vessel distribution. Values across the mapped area are normalized to sum to one, and hence are relative values and cannot be compared in absolute terms between species, only in terms of spatial patterns. For other species see Figs 7,6 and S6–S8.
(TIF)

S10 Fig Predicting baleen whale incidents. The relative risk of incidents plotted against the number of incidents per 1° grid cell for (a) all baleen, (b) blue, (c) fin, (d) humpback, (e) minke, (f) North Atlantic right, and (g) sei whales. Fitted regression line and estimates of variance explained are included.
(TIF)

S11 Fig Average sea surface salinity (SSS) (ppt) per 10km grid cell in the Northwest Atlantic. Values are shown from the (a) present day (1985–2015), (b) near-future (2035–2045), (c) and mid-future (2045–2055). Future projections made under 2x CO_2_ climate scenario. Data from the Community Earth System Model.
(TIF)

S12 Fig Average sea surface temperature (SST) (°C) per 10km grid cell in the Northwest Atlantic. Values are shown from the (a) present day (1985–2015), (b) near-future (2035–2045), (c) and mid-future (2045–2055) (c). Future projections made under 2x CO_2_ climate scenario. Data from the Community Earth System Model.
(TIF)

S13 Fig Average net primary productivity (NPP) (g C m^−2^ yr^−1^) per 10 km grid cell in the Northwest Atlantic. Values are shown from the (a) present day (1985–2015), (b) near-future (2035–2045), and (c) mid-future (2045–2055). Future projections made under 2x CO_2_ climate scenario. Data from the Community Earth System Model.
(TIF)

S14 Fig Depth of the ocean, displayed for the Northwest Atlantic. Data from GEBCO.
(TIF)
