# Supplementary material for: Assessing changing baleen whale distributions and reported incidents relative to vessel activity in the Northwest Atlantic
Source: PLoS One. 2025 Jan 15;20(1):e0315909. doi: 10.1371/journal.pone.0315909 (PMC11734950; doi:10.1371/journal.pone.0315909)
Supplement: S1 Table — Population estimates, Committee on the Status of Endangered Wildlife in Canada (COSEWIC) and Species at Risk Act (SARA) status, and year of designation of large baleen whale populations in the Northwest Atlantic (COSEWIC 2002, 2003, 2006, 2013, 2019a,b). (DOCX) [file pone.0315909.s001.docx]

**Table S1. COSEWIC and SARA Status and Population Estimates of Northwest Atlantic Large Baleen Whales.** Population estimates, Committee on the Status of Endangered Wildlife in Canada (COSEWIC) and Species at Risk Act (SARA) status, and year of designation of large baleen whale populations in the Northwest Atlantic [3-4, 21-23, 25].

| **Whale Species** | **Population Estimate** | **COSEWIC Status** | **Year COSEWIC Assessed** | **SARA Status** | **Year SARA Listed** |
| --- | --- | --- | --- | --- | --- |
| NA right whale | <400 | Endangered | 2013 | Endangered | 2005 |
| Blue whale Atlantic Population | <250 | Endangered | 2012 | Endangered | 2005 |
| Sei whale Atlantic Population | <1000 | Endangered | 2019 | Not Listed | N/A |
| Fin whale Atlantic Population | ~1,500 | Special Concern | 2019 | Special Concern | 2006 |
| Common Minke whale North Atlantic Subspecies | ~15,000 | Not at Risk | 2006 | Not Listed | N/A |
| Humpback whale Western North Atlantic Population | ~11,000 | Not at Risk | 2003 | Not Listed | N/A |
