# Supplementary material for: Assessing changing baleen whale distributions and reported incidents relative to vessel activity in the Northwest Atlantic
Source: PLoS One. 2025 Jan 15;20(1):e0315909. doi: 10.1371/journal.pone.0315909 (PMC11734950; doi:10.1371/journal.pone.0315909)
Supplement: S2 Table — Environmental variables of importance ranked by mean decrease accuracy (MDA) from the ensemble species distribution models for each species of baleen whale. 1 = variable of most importance, 6 = variable of least importance. SST refers to sea surface temperature, SSS refers to sea surface salinity, NPP refers to net primary productivity, and Bathy refers to bathymetry. (DOCX) [file pone.0315909.s002.docx]

**Table S2.** **Environmental variable ranking.** Environmental variables of importance ranked by mean decrease accuracy (MDA) from the ensemble species distribution models for each species of baleen whale. 1 = variable of most importance, 6 = variable of least importance. SST refers to sea surface temperature, SSS refers to sea surface salinity, NPP refers to net primary productivity, and Bathy refers to bathymetry.

| **Species** | **SST** | **SSS** | **NPP** | **Bathy** | **Shelf** | **Slope** |
| --- | --- | --- | --- | --- | --- | --- |
| Blue whale | 3 | 1 | 4 | 2 | 5 | 6 |
| Fin whale | 2 | 1 | 3 | 4 | 5 | 6 |
| Humpback whale | 1 | 2 | 4 | 3 | 5 | 6 |
| Minke whale | 2 | 1 | 4 | 3 | 5 | 6 |
| NA right whale | 2 | 1 | 3 | 4 | 5 | 6 |
| Sei whale | 2 | 1 | 4 | 3 | 5 | 6 |
