# Supplementary material for: Assessing changing baleen whale distributions and reported incidents relative to vessel activity in the Northwest Atlantic
Source: PLoS One. 2025 Jan 15;20(1):e0315909. doi: 10.1371/journal.pone.0315909 (PMC11734950; doi:10.1371/journal.pone.0315909)
Supplement: S3 Table — This table delineates the average habitat suitability values across different species: Blue, fin, and humpback whales, compared between areas of presence and absence, and for sei whales, compared across locations with varying frequencies of detections (only a high-detection and low-detection example has been provided). (DOCX) [file pone.0315909.s003.docx]

**Table S3. Comparative Analysis of Habitat Suitability in Relation to Acoustic Whale Detections.** This table delineates the average habitat suitability values across different species: blue, fin, and humpback whales, compared between areas of presence and absence, and for sei whales, compared across locations with varying frequencies of detections (only a high-detection and low-detection example has been provided).

| **Species** | **Average Habitat Suitability** | |
| --- | --- | --- |
|  | **Presence** | **Absence** |
| Blue whale | 264.84 | 224.61 |
| Fin whale | 395.94 | 352.23 |
| Humpback whale | 548.56 | 362.92 |
|  | **Number of Detections** | **Average Habitat Suitability** |
| Sei whale | 88 | 9.07 |
| Sei whale | 271 | 533.49 |
