# Supplementary figures and images for: Assessing changing baleen whale distributions and reported incidents relative to vessel activity in the Northwest Atlantic
Source: PLoS One. 2025 Jan 15;20(1):e0315909. doi: 10.1371/journal.pone.0315909 (PMC11734950; doi:10.1371/journal.pone.0315909)

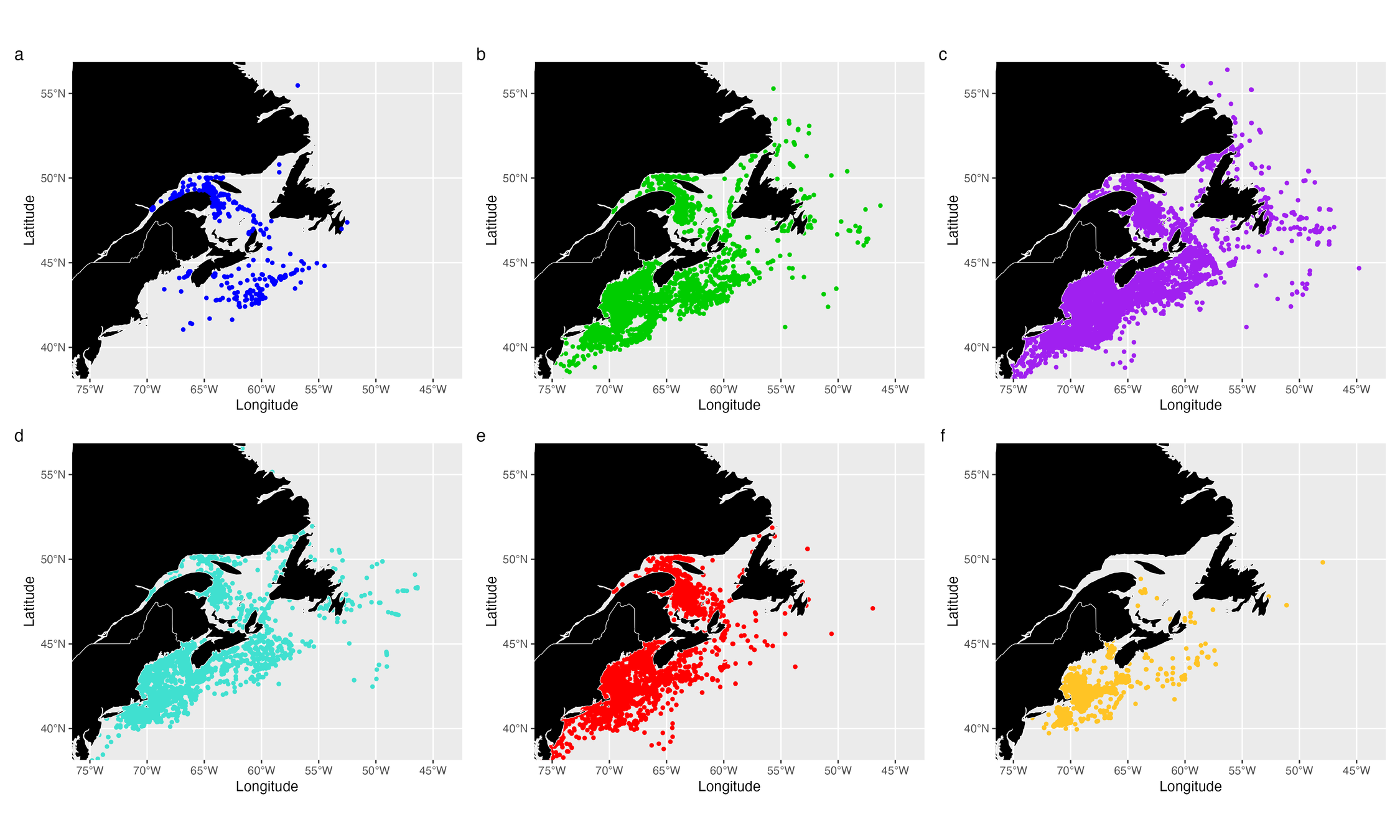

Supplement: S1 Fig — Shown are reported sightings for the (a) blue whale, (b) fin whale, (c) humpback whale, (d) minke whale, (e) North Atlantic right whale, and (f) sei whale. Data provided by DFO-Maritimes opportunistic sightings database and the Whitehead Lab, the North Atlantic Right Whale Consortium, Environment Canada Seabirds at Sea, the Réseau D’observation de Mammifères Marins, and the Ocean Biodiversity Information System. (TIF) [file pone.0315909.s004.tif]

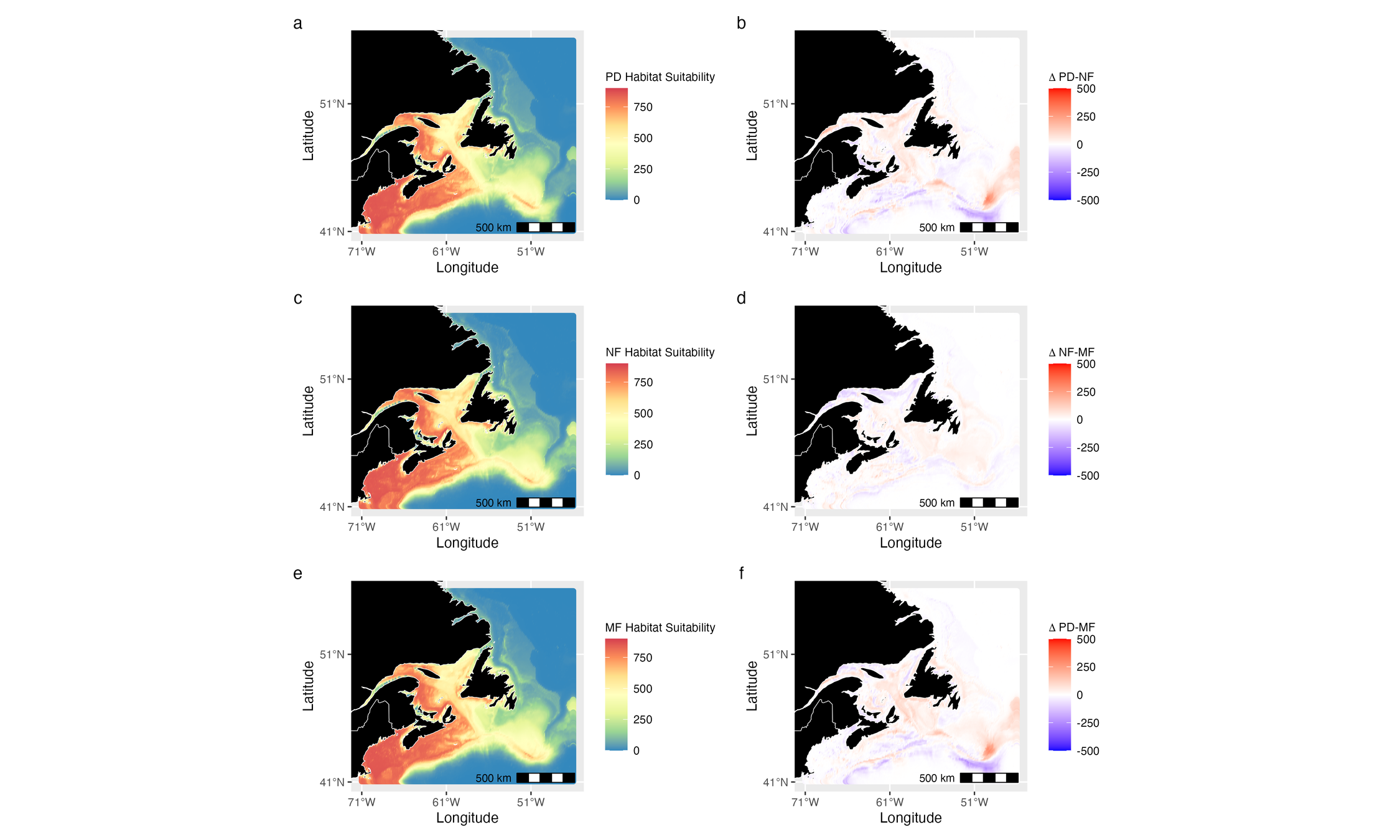

Supplement: S2 Fig — Projections from an ensemble species distribution model show (a) present-day (PD) habitat suitability (1985–2015). (b) Projected change in suitability from the present day to near-future (NF). (c) Near-future habitat suitability (2035–2045). (d) Change in habitat suitability from the near to mid-future (MF). (e) Mid-future habitat suitability (2045–2055). (f) Change in habitat suitability from the present day to the mid-future. Future projections refer to a climate scenario assuming a doubling of CO2 concentrations. Red colours reflect high habitat suitability values (HSV) and blue colours reflect areas with lower habitat suitability. Habitat suitability values reflect spring, summer and fall, but not winter suitability. For other species see Figs 4,5 and S3–S5 Figs. (TIF) [file pone.0315909.s005.tif]

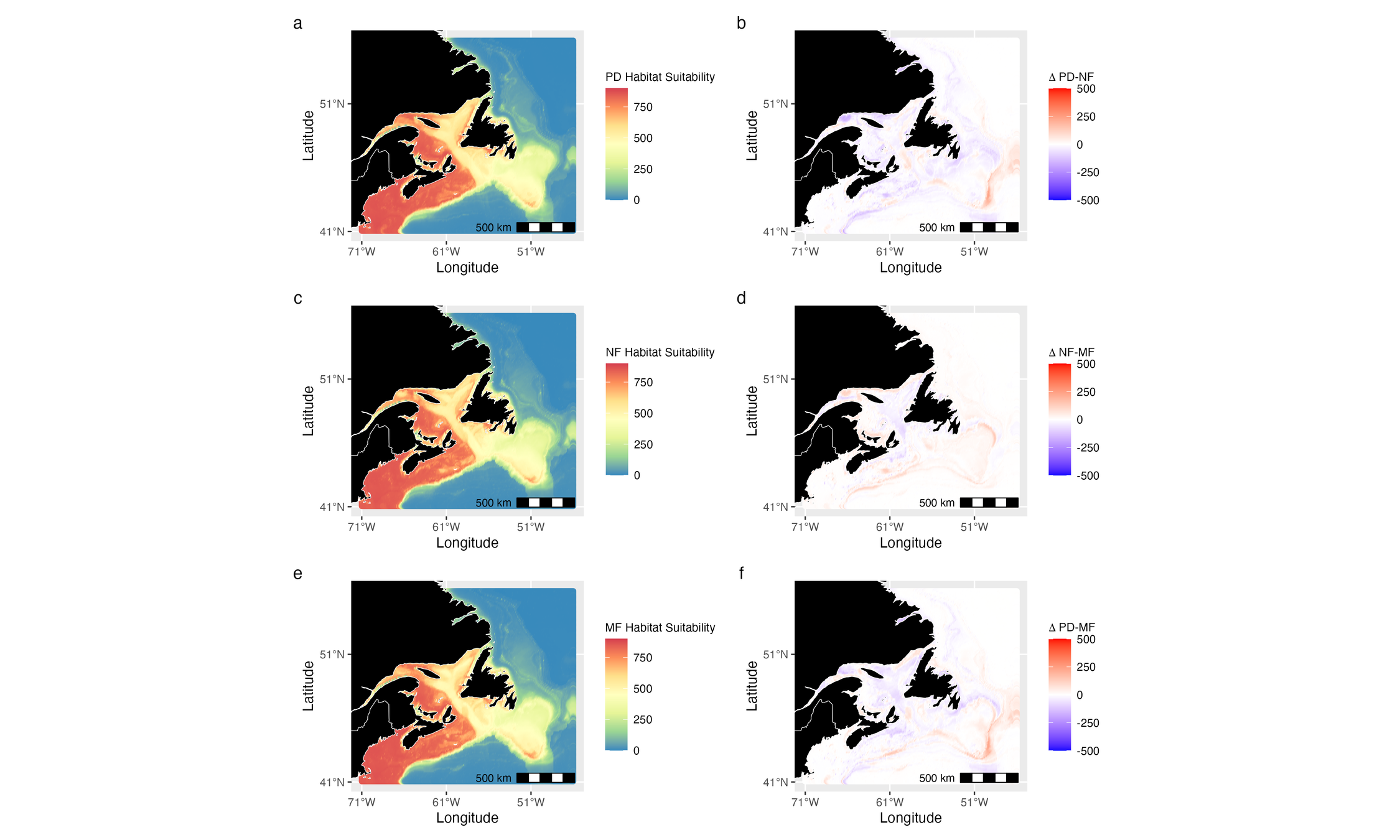

Supplement: S3 Fig — Projections from an ensemble species distribution model show a) present-day (PD) habitat suitability (1985–2015). (b) Projected change in suitability from the present day to near-future (NF). (c) Near-future habitat suitability (2035–2045). (d) Change in habitat suitability from the near to mid-future (MF). (e) Mid-future habitat suitability (2045–2055). (f) Change in habitat suitability from the present day to the mid-future. Future projections refer to a climate scenario assuming a doubling of CO2 concentrations. Red colours reflect high habitat suitability values (HSV) and blue colours reflect areas with lower habitat suitability. Habitat suitability values reflect spring, summer and fall, but not winter suitability. For other species see Figs 4,5 and S2–S5. (TIF) [file pone.0315909.s006.tif]

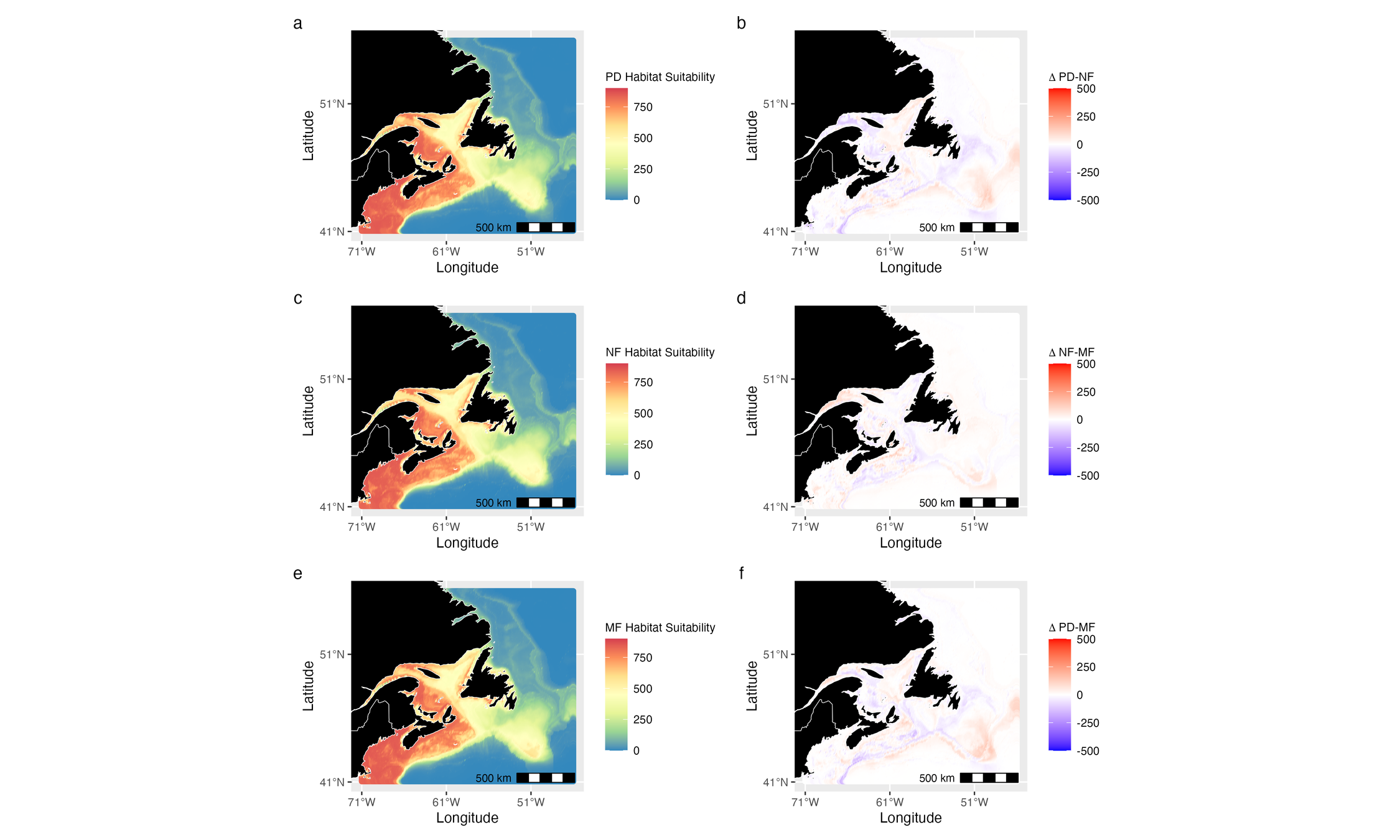

Supplement: S4 Fig — Projections from an ensemble species distribution model show a) present-day (PD) habitat suitability (1985–2015). (b) Projected change in suitability from the present day to near-future (NF). (c) Near-future habitat suitability (2035–2045). (d) Change in habitat suitability from the near to mid-future (MF). (e) Mid-future habitat suitability (2045–2055). (f) Change in habitat suitability from the present day to the mid-future. Future projections refer to a climate scenario assuming a doubling of CO2 concentrations. Red colours reflect high habitat suitability values (HSV) and blue colours reflect areas with lower habitat suitability. Habitat suitability values reflect spring, summer and fall, but not winter suitability. For other species see Figs 4,5 and S2–S5. (TIF) [file pone.0315909.s007.tif]

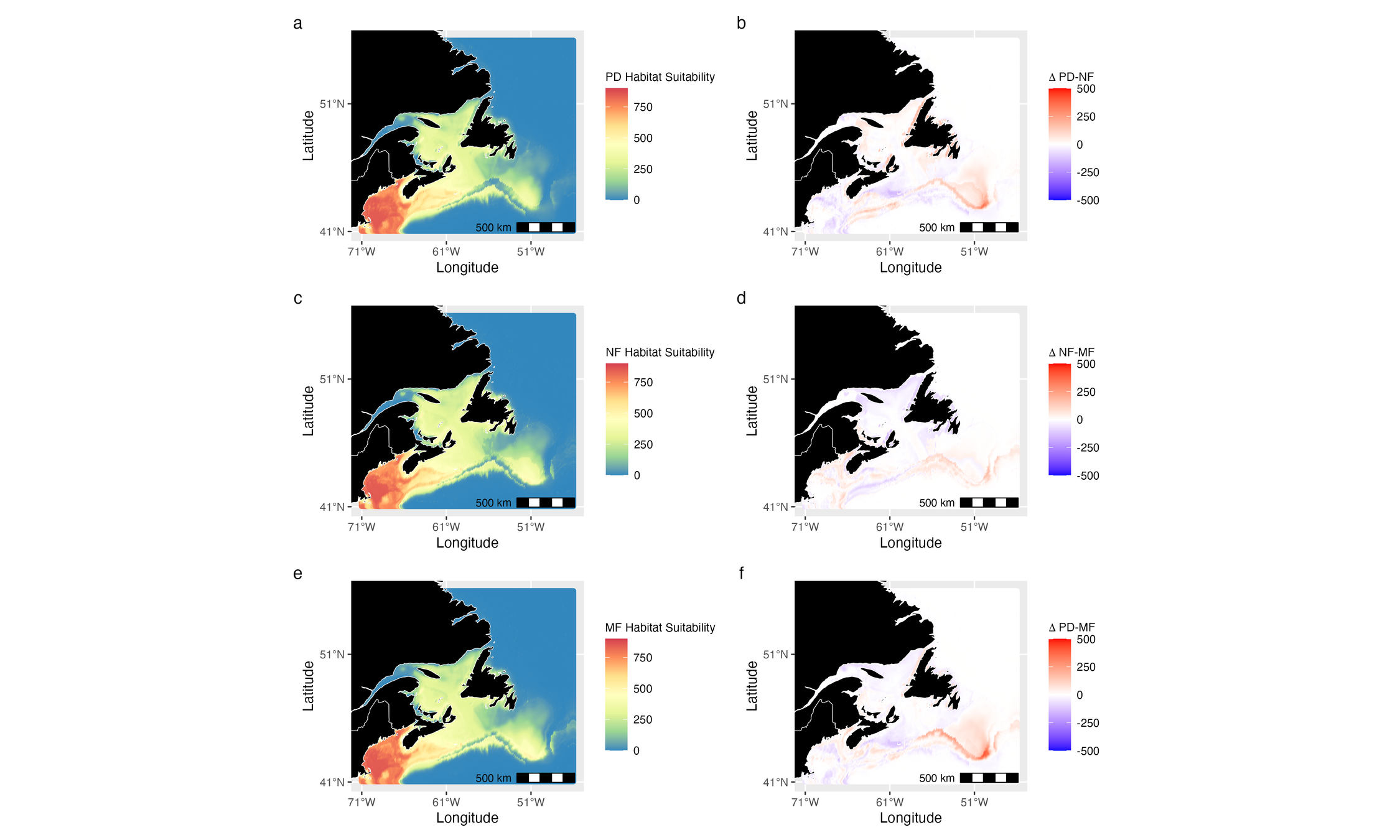

Supplement: S5 Fig — Projections from an ensemble species distribution model show (a) present-day (PD) habitat suitability (1985–2015). (b) Projected change in suitability from the present day to near-future (NF). (c) Near-future habitat suitability (2035–2045). (d) Change in habitat suitability from the near to mid-future (MF). (e) Mid-future habitat suitability (2045–2055). (f) Change in habitat suitability from the present day to the mid-future. Future projections refer to a climate scenario assuming a doubling of CO2 concentrations. Red colours reflect high habitat suitability values (HSV) and blue colours reflect areas with lower habitat suitability. Habitat suitability values reflect spring, summer and fall, but not winter suitability. For other species see Figs 4,5 and S2–S5. (TIF) [file pone.0315909.s008.tif]

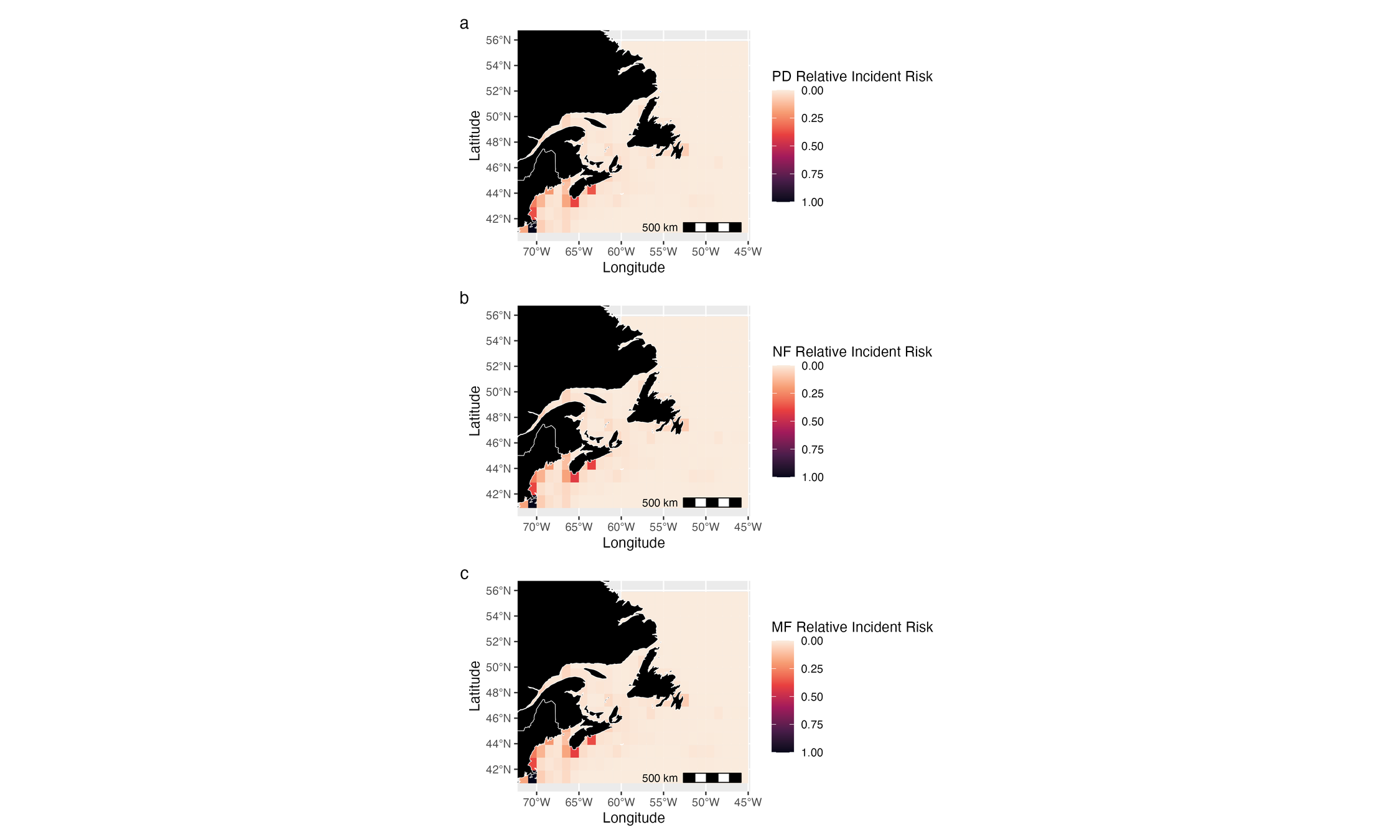

Supplement: S6 Fig — Relative incident risk (a) for the present day (PD) (1985–2015) for all vessels, (b) for the near-future (NF) (2035–2045), and (c) mid-future (MF) (2045–2055) under climate scenario 2x CO2. Darker colors indicate areas where fin whales are predicted to be more vulnerable to incidents based on species and vessel distribution. Values across the mapped area are normalized to sum to one, and hence are relative values and cannot be compared in absolute terms between species, only in terms of spatial patterns. For other species see Figs 7, 6 and S7–S9 . (TIF) [file pone.0315909.s009.tif]

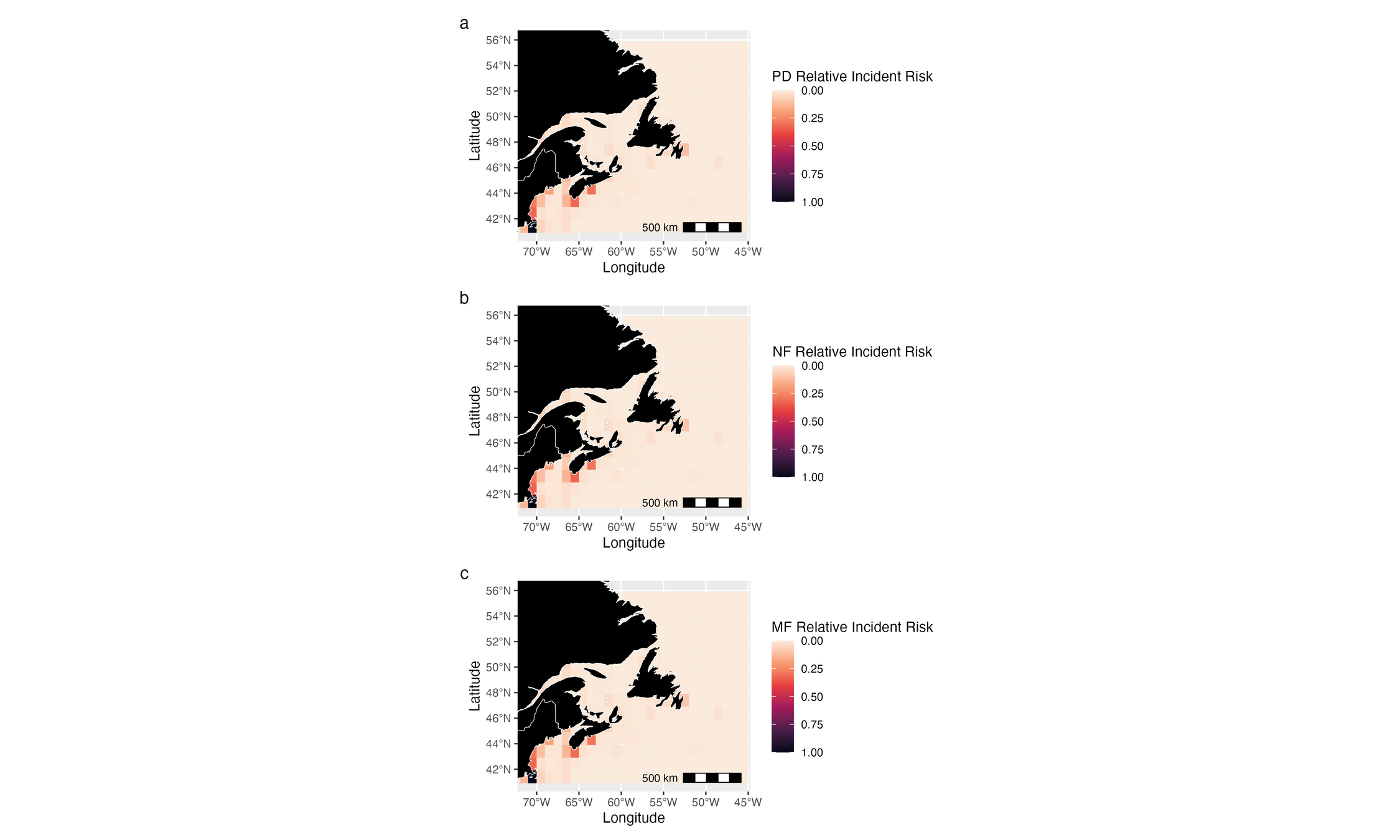

Supplement: S7 Fig — Relative incident risk (a) for the present day (PD) (1985–2015) for all vessels, (b) for the near-future (NF) (2035–2045), and (c) mid-future (MF) (2045–2055) under climate scenario 2x CO2. Darker colors indicate areas where humpback whales are predicted to be more vulnerable to incidents based on species and vessel distribution. Values across the mapped area are normalized to sum to one, and hence are relative values and cannot be compared in absolute terms between species, only in terms of spatial patterns. For other species see Figs 7, 6, S6, S8 and S9. (TIF) [file pone.0315909.s010.tif]

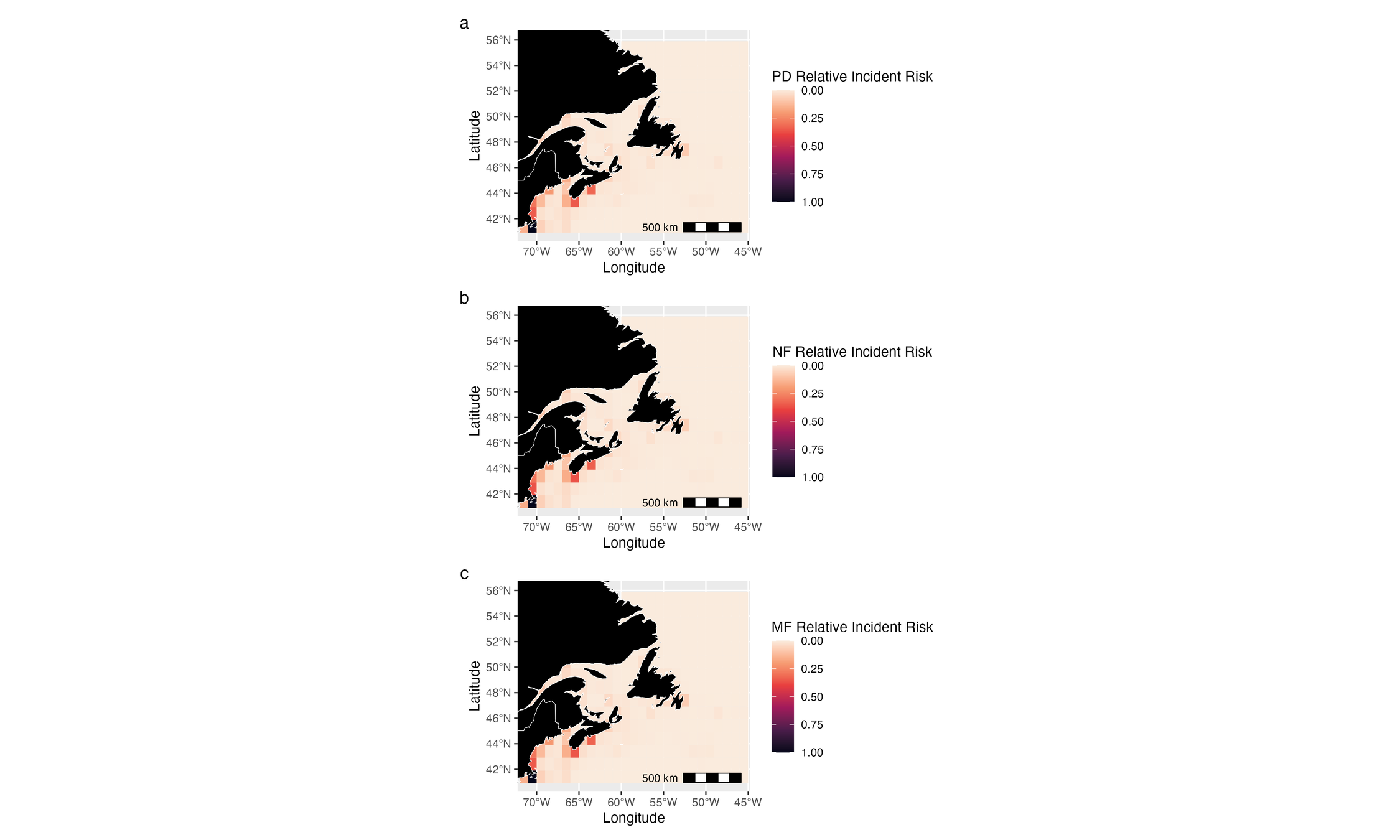

Supplement: S8 Fig — Relative incident risk (a) for the present day (PD) (1985–2015) for all vessels, (b) for the near-future (NF) (2035–2045), and (c) mid-future (MF) (2045–2055) under climate scenario 2x CO2. Darker colors indicate areas where minke whales are predicted to be more vulnerable to incidents based on species and vessel distribution. Values across the mapped area are normalized to sum to one, and hence are relative values and cannot be compared in absolute terms between species, only in terms of spatial patterns. For other species see Figs 7,6, S6, S7 and S9. (TIF) [file pone.0315909.s011.tif]

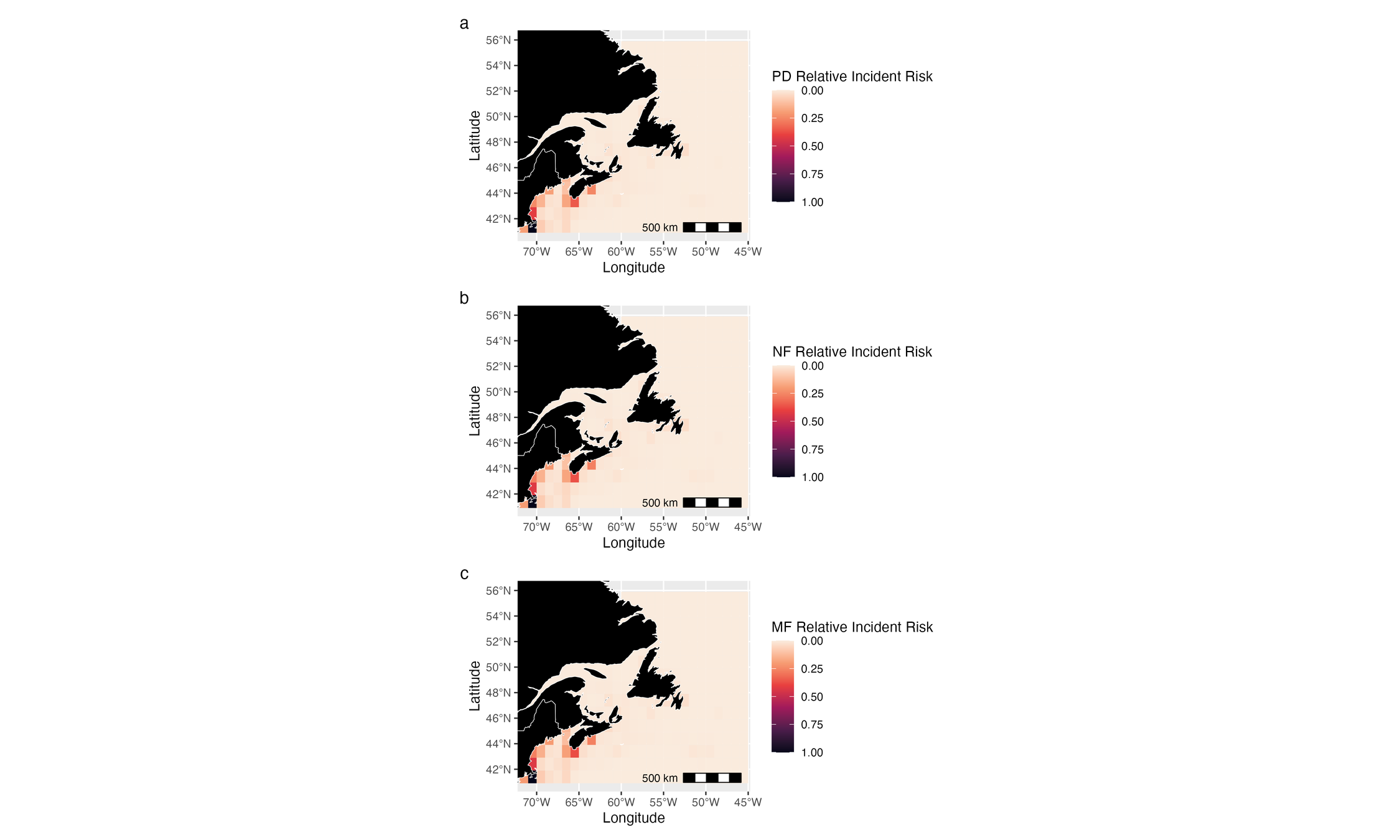

Supplement: S9 Fig — Relative incident risk (a) for the present day (PD) (1985–2015) for all vessels, (b) for the near-future (NF) (2035–2045), and (c) mid-future (MF) (2045–2055) under climate scenario 2x CO2. Darker colors indicate areas where sei whales are predicted to be more vulnerable to incidents based on species and vessel distribution. Values across the mapped area are normalized to sum to one, and hence are relative values and cannot be compared in absolute terms between species, only in terms of spatial patterns. For other species see Figs 7,6 and S6–S8. (TIF) [file pone.0315909.s012.tif]

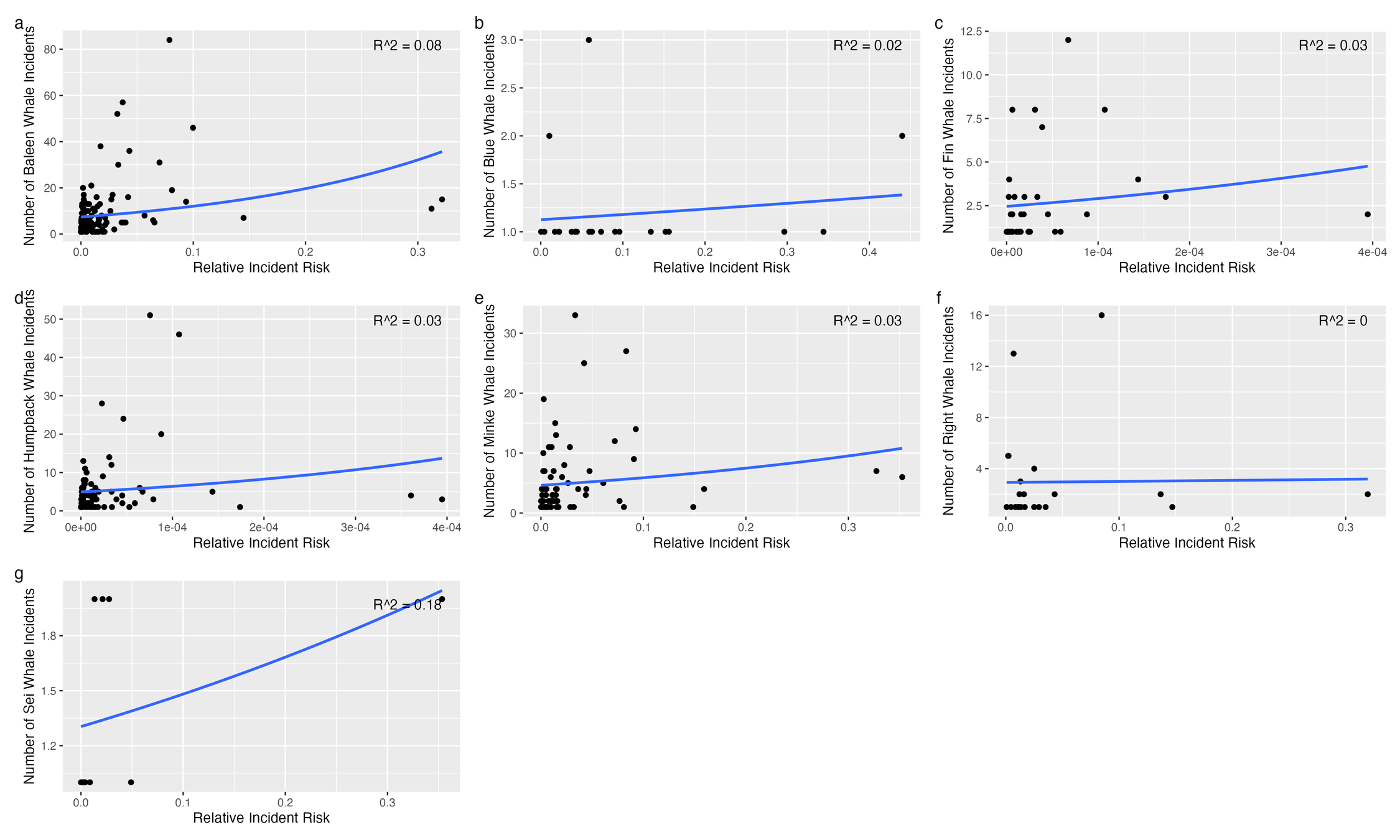

Supplement: S10 Fig — The relative risk of incidents plotted against the number of incidents per 1° grid cell for (a) all baleen, (b) blue, (c) fin, (d) humpback, (e) minke, (f) North Atlantic right, and (g) sei whales. Fitted regression line and estimates of variance explained are included. (TIF) [file pone.0315909.s013.tif]

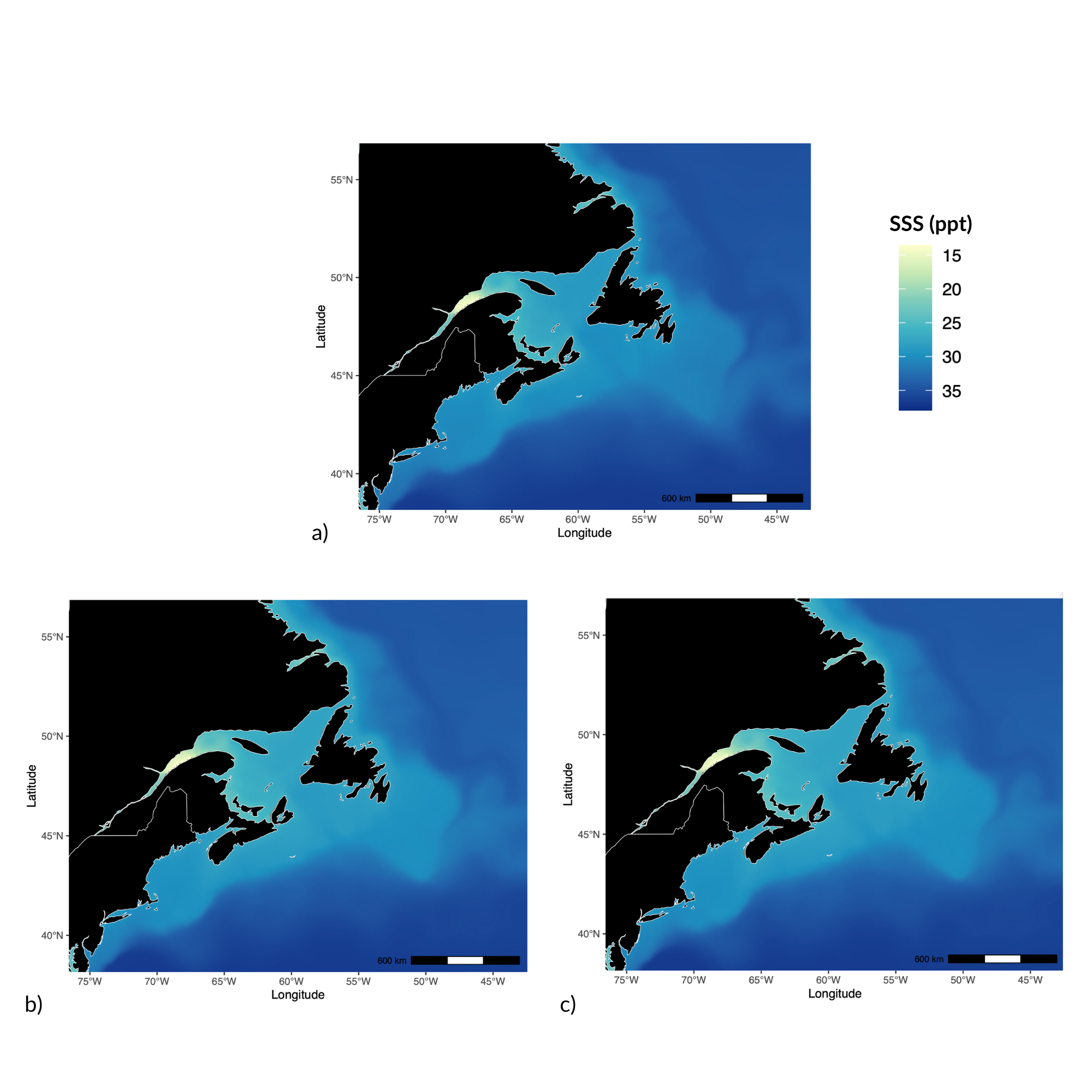

Supplement: S11 Fig — Values are shown from the (a) present day (1985–2015), (b) near-future (2035–2045), (c) and mid-future (2045–2055). Future projections made under 2x CO2 climate scenario. Data from the Community Earth System Model. (TIF) [file pone.0315909.s014.tif]

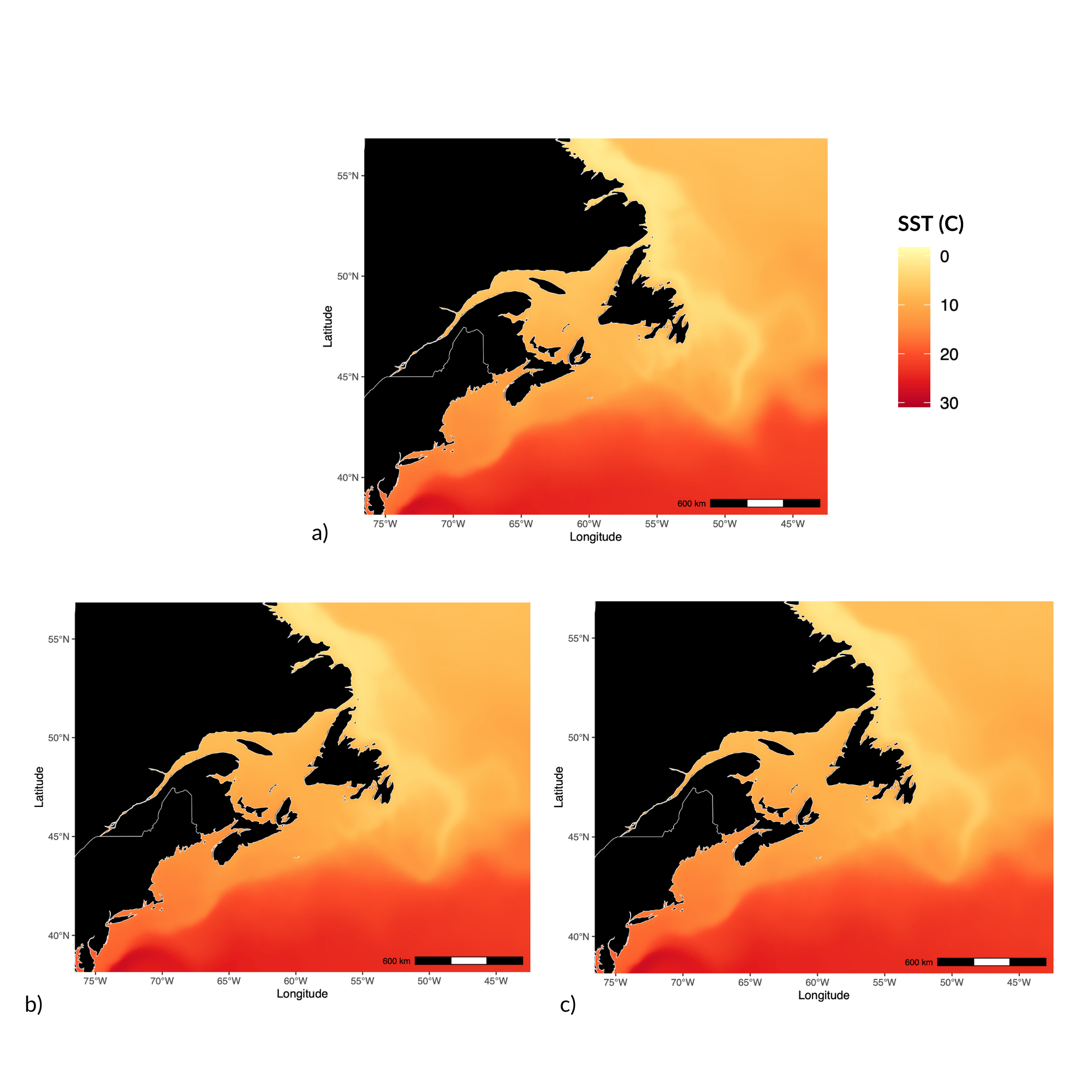

Supplement: S12 Fig — Values are shown from the (a) present day (1985–2015), (b) near-future (2035–2045), (c) and mid-future (2045–2055) (c). Future projections made under 2x CO2 climate scenario. Data from the Community Earth System Model. (TIF) [file pone.0315909.s015.tif]

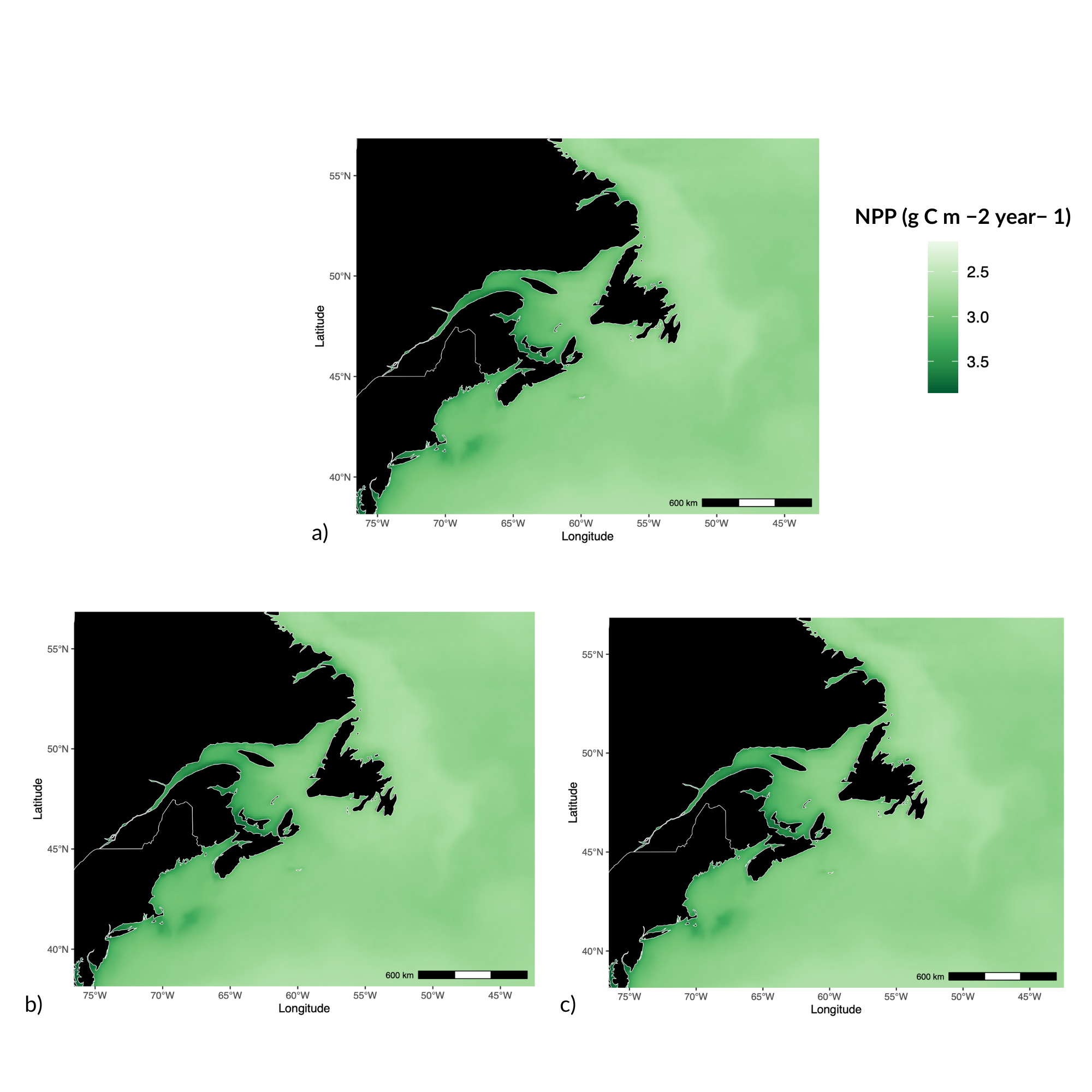

Supplement: S13 Fig — Values are shown from the (a) present day (1985–2015), (b) near-future (2035–2045), and (c) mid-future (2045–2055). Future projections made under 2x CO2 climate scenario. Data from the Community Earth System Model. (TIF) [file pone.0315909.s016.tif]

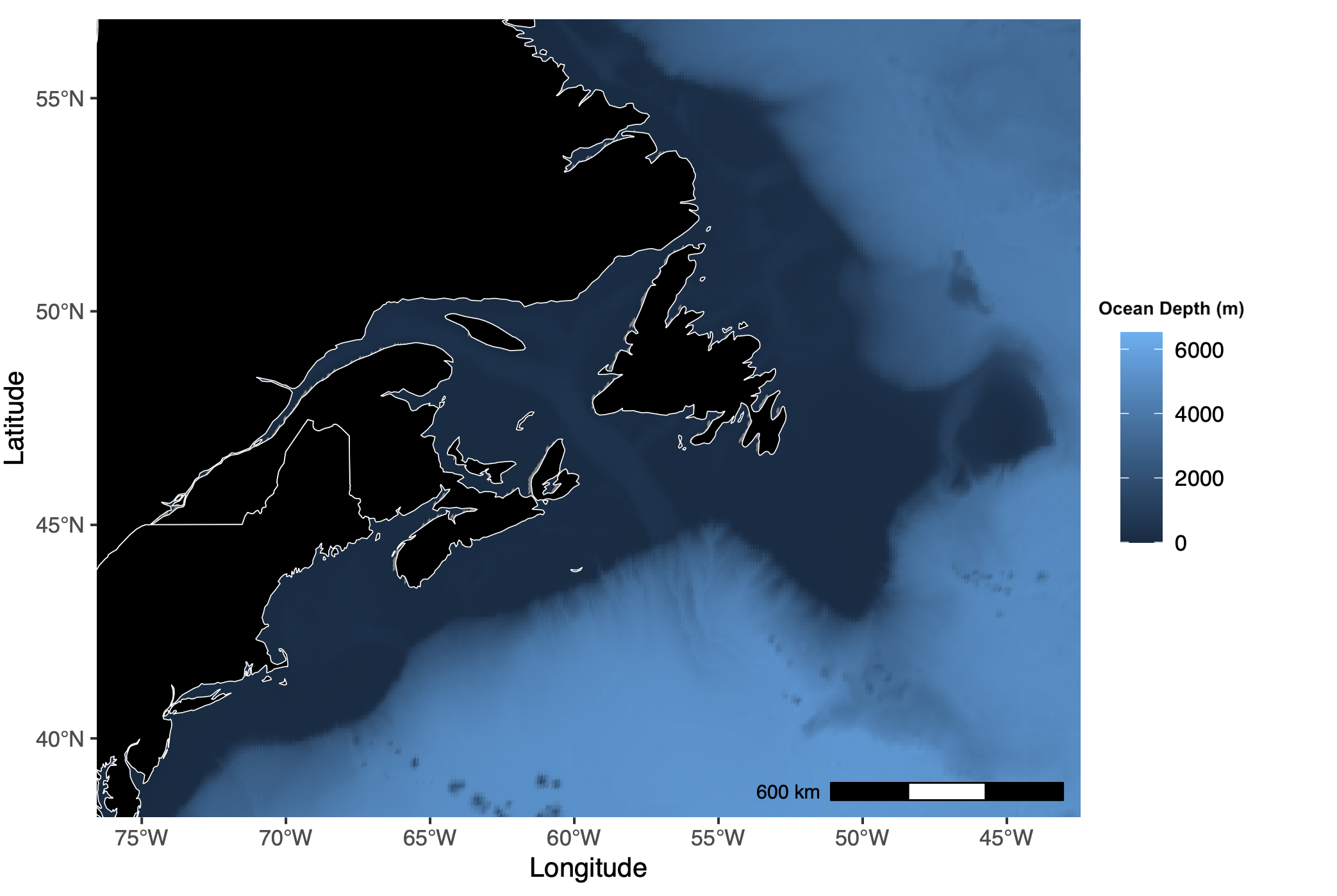

Supplement: S14 Fig — Data from GEBCO. (TIF) [file pone.0315909.s017.tif]
